# NKp46‐specific single domain antibodies enable facile engineering of various potent NK cell engager formats

**DOI:** 10.1002/pro.4593

**Published:** 2023-02-24

**Authors:** Britta Lipinski, Paul Arras, Lukas Pekar, Daniel Klewinghaus, Ammelie Svea Boje, Simon Krah, Jasmin Zimmermann, Katja Klausz, Matthias Peipp, Vanessa Siegmund, Andreas Evers, Stefan Zielonka

**Affiliations:** ^1^ Protein Engineering and Antibody Technologies Merck Healthcare KGaA Darmstadt Germany; ^2^ Institute for Organic Chemistry and Biochemistry Technical University of Darmstadt Darmstadt Germany; ^3^ Division of Antibody‐Based Immunotherapy, Department of Internal Medicine II University Hospital Schleswig‐Holstein and Christian‐Albrechts‐University Kiel Kiel Germany; ^4^ Protein and Cell Sciences Merck Healthcare KGaA Darmstadt Germany; ^5^ Computational Chemistry and Biology Merck Healthcare KGaA Darmstadt Germany

**Keywords:** ADCC, antibody engineering, bispecific antibody, multifunctional antibody, NK cell engager, NK cell redirection, NKp46, single domain antibody, valencies, VHH

## Abstract

Herein, we describe the generation of potent NK cell engagers (NKCEs) based on single domain antibodies (sdAbs) specific for NKp46 harboring the humanized Fab version of Cetuximab for tumor targeting. After immunization of camelids, a plethora of different VHH domains were retrieved by yeast surface display. Upon reformatting into Fc effector‐silenced NKCEs targeting NKp46 and EGFR in a strictly monovalent fashion, the resulting bispecific antibodies elicited potent NK cell‐mediated killing of EGFR‐overexpressing tumor cells with potencies (EC_50_killing) in the picomolar range. This was further augmented via co‐engagement of Fcγ receptor IIIa (FcγRIIIa). Importantly, NKp46‐specific sdAbs enabled the construction of various NKCE formats with different geometries and valencies which displayed favorable biophysical and biochemical properties without further optimization. By this means, killing capacities were further improved significantly. Hence, NKp46‐specific sdAbs are versatile building blocks for the construction of different NKCE formats.

## 1 INTRODUCTION

Bispecific antibodies (bsAbs) have proven to be of utmost relevance for disease treatment, especially for indications in oncology (Krah et al., 2018, 2017; Labrijn et al., 2019). As of July 12, 2022 (www.antibodysociety.org) a total number of seven bispecific antibodies have been approved for therapy either in the United States or Europe (of note, Catumaxomab was withdrawn in 2017 (Brinkmann & Kontermann, 2021)) with two more molecules in regulatory review. Moreover, around 200 bsAbs are currently assessed in clinical trials (Carter & Rajpal, 2022). One very promising field for bsAbs relies on the conditional agonism of activating receptors or costimulatory receptors on immune cell populations. In this respect, recruiting T cells via bsAbs referred to as T cell engagers show great promise, especially for hematological malignancies but also for the treatment of melanoma (Assouline et al., 2020; Killock, 2021; Zhao et al., 2019). In recent years, also other immune cell subsets gained substantial interest for being exploited as effector population such as the redirection of natural killer cells (NK cells) (Demaria et al., 2021; Hu et al., 2019; Huntington et al., 2020).

NK cells are part of the early host defense in the body, having the natural capacity to distinguish between healthy tissues and stressed or diseased cells. This is due to a complex interplay between several distinct germline‐encoded activating and inhibitory receptors (Chiossone et al., 2018; Gonzalez‐Rodriguez & Sordo‐Bahamonde, 2019). Inhibitory receptors such as natural killer group 2A (NKG2A) or killer‐immunoglobulin‐like receptors (KIRs) recognize “self” ligands normally expressed by host cells (Carlsten & Järås, 2019; Vivier et al., 2008). In addition, NK cells express an array of activating receptors, for instance, the natural cytotoxicity receptors (NCRs), NKG2D or DNAM‐1 (Koch & Tesar, 2017; Morgado et al., 2011). Ligands of those receptors are typically upregulated on stressed cells, eventually resulting in NK cell activation. However, shedding of ligands for activating receptors has been described as one mechanism of tumor immune escape (Reiners et al., 2013; Schlecker et al., 2014; Wang et al., 2014). Moreover, tumor cells might downregulate ligand‐derived danger signals or upregulate inhibitory human leukocyte antigen (HLA) molecules and consequently evade immune recognition by NK cells (Balsamo et al., 2012).

Furthermore, NK cells might become activated in an antibody‐directed fashion. Triggering of the low affinity FcγRIIIa (CD16a) by target cells opsonized with antibodies causes efficient NK cell activation resulting in degranulation and target cell eradication. This process, referred to as antibody‐dependent cell‐mediated cytotoxicity (ADCC) is considered as one important mode of action of many therapeutic antibodies (Beano et al., 2008; Darwich et al., 2021; Seidel et al., 2013; Wang et al., 2017). Yet, the capability of an antibody to elicit ADCC is affected in several ways, for instance, by antigen densities on target cells, FcγRIIIa polymorphism or competition with serum IgG (Bibeau et al., 2009; Koch & Tesar, 2017; Preithner et al., 2006). To overcome these inherent limitations of classical antibody therapies, bi‐ and multi‐specific NKCEs have been developed, in which one paratope binds to FcγRIIIa with high affinity, while the other paratope is directed against a tumor antigen (Koch & Tesar, 2017; Rothe et al., 2015; Wingert et al., 2021). Several of FcγRIIIa‐specific NKCEs are currently investigated in clinical trials (Bartlett et al., 2020; Demaria et al., 2021). Another route that is presently pursued relies on targeting the array of activating NK cell receptors for the construction of potent NKCEs (Peipp et al., 2022). This has been accomplished in several different ways. For instance, bifunctional immunoligands have been described in which a tumor‐associated antigen (TAA)‐directed paratope was fused to the extracellular region of a ligand for an activating receptor or to affinity‐optimized versions thereof (Peipp et al., 2015; Pekar et al., 2021; von Strandmann et al., 2006). Moreover, bispecific or multifunctional NKCEs were constructed that bridge a TAA on tumor cells with an activating receptor on NK cells such as NKG2D or NKp30 (Colomar‐Carando et al., 2022; Klausz et al., 2022; Klewinghaus et al., 2022; Raynaud et al., 2021). In one of the most prominent examples, Vivier and colleagues engineered trifunctional NKCEs based on the engagement of two NK cell activating receptors, that is, NKp46 as well as FcγRIIIa, for very potent effector cell redirection (Gauthier et al., 2019). Of note, NKCEs based on this approach are currently assessed in early‐stage clinical trials (e.g., NCT05086315) (Gauthier et al., 2021).

**FIGURE 1:**
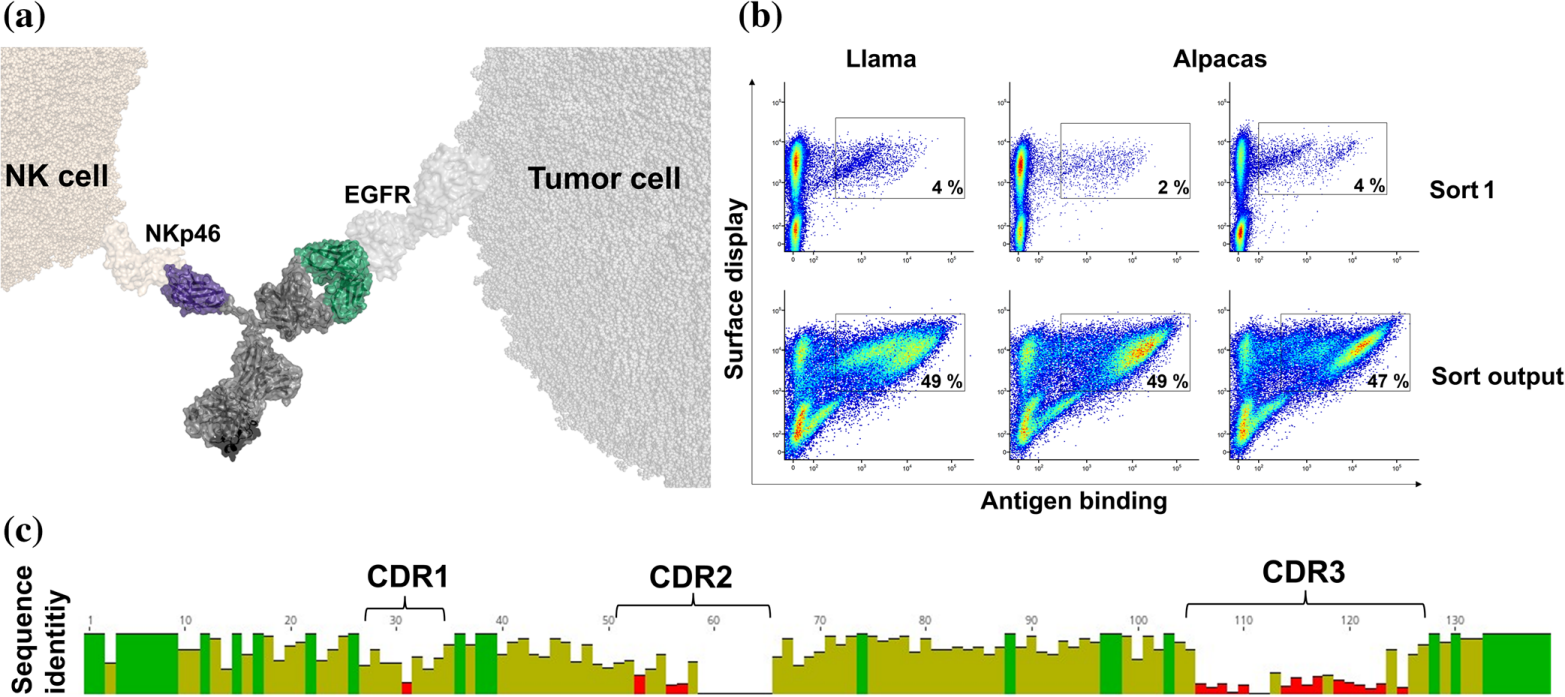
Camelid immunization combined with YSD allows for the generation of a diverse panel of NKp46‐specific sdAbs for NK cell redirection. (a) Schematic depiction of a bispecific SEEDbody for NK redirection based on a NKp46‐specific VHH molecule in combination with a humanized version of the Fab arm of Cetuximab for EGFR targeting. The scheme was generated using PyMol software version 2.3.0. (b) FACS‐based selection for the isolation of NKp46‐specific VHHs by utilization of a two‐dimensional staining strategy for full‐length VHH display and NKp46 binding at a concentration of 1 μM. Of note, plots in the upper row show representative 10^6^ cells of the sort, while plots in the lower row show representative 5 × 10^4^ cells of the corresponding sorting output in order to visualize enrichment. (c) Graphical alignment of unique amino acid sequences of 40 VHH clones obtained from YSD library sorting. CDRs are indicated. Red bars represent high sequence diversity and green bars indicate high sequence conservation at a given position. Alignment obtained using MUSCLE alignment with Geneious Prime® January 1, 2021 software.

In this work, we have engineered EGFR‐specific NKCEs triggering NKp46‐mediated tumor cell eradication by employing camelid‐derived single domain antibodies (sdAbs) (Figure 1a). After immunization of camelids, NKp46‐specific VHH domains were isolated using yeast surface display (YSD) (Roth et al., 2020; Valldorf et al., 2022). Bispecific NKCEs harboring NKp46‐directed sdAbs elicited efficient NK cell‐mediated killing of EGFR‐positive tumor cells. In addition, we demonstrate that killing capacities of NKCEs based on NKp46‐specific VHH domains can be significantly augmented by protein engineering.

## 2 RESULTS

### 2.1 Immunization of camelids followed by YSD enables the isolation of a diverse panel of NKp46‐specific VHH sdAbs

In order to generate bsAbs triggering NK cells via NCR NKp46 we aimed at isolating sdAbs from camelids, since it is well established that camelid‐derived VHH domains afford the benefit of multiple engineering options (Chanier & Chames, 2019; Pekar et al., 2020). To this end, two Alpacas (*Vicugna pacos*) and one Llama (*Lama glama*) were immunized with the recombinant extracellular region of human NKp46. Subsequently, for each specimen a YSD library was constructed and selected by FACS. Sorting was conducted in a two‐dimensional manner, to simultaneously detect for functional VHH surface expression as well as NKp46 binding. In this respect, approximately 10^8^ cells per library were sorted (Figure 1b, upper panel). For this, an antigen concentration of 1 μM was used, to also enrich for clones potentially displaying lower affinities. Intriguingly, for each of the libraries we already observed a distinct antigen‐binding population of 2%–4%. Subsequently, the sorting output was reanalyzed to get a glimpse about the enrichment, revealing more than 50% of antigen‐binding cells in the FACS‐sorted population (Figure 1b, lower panel). From each library 96 clones (288 clones in total) were sequenced and revealed a panel of 199 unique sdAbs. Based on clonotyping (a sequence identity of >90% within CDR3 was defined as the same clonotype), we selected 40 clones for bsAb expression, each representing a unique clonotype (Figure 1c). In addition to sequence diversity of the VHH domains, most sequences revealed a considerable human‐likeness, a low number of potential chemical degradation sites and post‐translational modification sites as well as in general adequate in silico physico‐chemical properties (Table SI). In more detail, sequence identity compared to the most similar human germline ranged between 63.4% for NKp46.40  as well as NKp46.35  and 80.5% for NKp46.1 if calculated for the complete sdAbs. Moreover, only a few sequences exhibited cysteines in noncanonical positions, such as NKp46.30, NKp46.36 and NKp46.38, all harboring two cysteines presumably forming an additional disulfide bond. Of note, only NKp46.37 displayed an unpaired cysteine residue that might potentially cause issues during manufacturing. Additionally, most of the screened VHHs revealed only a low number of amino acid residues considered as susceptible for potential (bio)chemical alterations, that is, methionine oxidization, asparagine and glutamine deamidation as well as amino acid isomerization and *N‐*glycosylation. Moreover, only two sequences, NKp46.32 and NKp46.33, showed considerable surface hydrophobicity (aggregation score). Finally, the set of identified clones displayed a broad coverage of computed isoelectric point (p*I*) values ranging from pH 3.2 up to pH 9.4. Due to these overall favorable developability properties determined in silico, these VHHs represent promising starting points for potential lead optimization studies.

For bsAb construction the strand‐exchanged engineered domain (SEED) heterodimerization platform was applied that relies on beta‐strand exchanges of IgG and IgA isotypes, resulting in preferential heavy chain heterodimerization (Davis et al., 2010). Each of the VHH domains was genetically engrafted onto the hinge region of the AG chain of the SEEDbody, whereas the Fab region of a humanized version of Cetuximab (hu225) was fused to the GA chain. The RF mutation was introduced into the GA chain in order to obviate purification of GA:GA homodimers that might form during expression (Tustian et al., 2016). Of note, an effector‐silenced (eff−) version of the Fc region of the SEEDbody was used to solely focus on killing capacities mediated by the isolated NKp46‐specific sdAb. Expression yields were in the triple digit milligram‐per‐liter scale for the vast majority of NKp46 SEEDbodies eff−, generally indicating adequate productivities for transient expression (Table 1) (Pekar et al., 2021, 2020). Besides, also aggregation propensities as determined by analytical size exclusion chromatography (SEC) post protein A purification indicated favorable biophysical properties of the herein engineered NKp46 SEEDbodies eff−. In this respect, SEC profiles for 37 out of 40 molecules were above 90% target monomer peak, except for NKp46.13 SEEDbody eff− with 87.1% target monomer peak, NKp46.20 SEEDbody eff− with 83.9% main peak purity and NKp46.29 SEEDbody eff− with 81.8% target monomer peak (Table 1).

**TABLE 1:** Biochemical and biophysical properties of VHH‐based NKCEs targeting NKp46 and EGFR.

#	Yield (mg/L)	SEC (%)	KD (M)	*k*_on_ (1/Ms)	*k*_off_ (1/s)
NKp46.1 SEEDbody eff−	190	94.6	3.84E−09	6.07E+05	2.33E−03
NKp46.2 SEEDbody eff−	176; 176^a^	94.4; 92.1^a^	1.05E−09	2.85E+05	2.97E−04
NKp46.3 SEEDbody eff−	147	96.1	1.71E−09	3.06E+05	5.24E−04
NKp46.4 SEEDbody eff−	180	94.7	1.20E−09	6.32E+05	7.55E−04
NKp46.5 SEEDbody eff−	234	96.3	5.01E−10	6.48E+05	3.25E−04
NKp46.6 SEEDbody eff−	228	94.8	4.42E−09	1.05E+05	4.62E−04
NKp46.7 SEEDbody eff−	200	92.9	6.89E−09	1.48E+05	1.02E−03
NKp46.8 SEEDbody eff−	175	91	1.34E−09	5.89E+05	7.90E−04
NKp46.9 SEEDbody eff−	166	93.3	1.43E−09	6.85E+05	9.77E−04
NKp46.10 SEEDbody eff−	168	96.8	2.65E−10	9.41E+04	2.50E−05
NKp46.11 SEEDbody eff−	172	97.9	2.31E−09	2.63E+05	6.07E−04
NKp46.12 SEEDbody eff−	165	94.9	4.09E−09	5.56E+05	2.27E−03
NKp46.13 SEEDbody eff−	181	87.1	1.87E−09	5.78E+05	1.08E−03
NKp46.14 SEEDbody eff−	183	96.7	1.94E−09	2.90E+05	5.63E−04
NKp46.15 SEEDbody eff−	171	96.3	5.34E−09	2.96E+05	1.58E−03
NKp46.16 SEEDbody eff−	170	93.4	1.05E−08	3.04E+05	3.19E−03
NKp46.17 SEEDbody eff−	141	96.7	1.85E−08	6.25E+04	1.16E−03
NKp46.18 SEEDbody eff−	177	93.6	6.85E−09	2.44E+05	1.67E−03
NKp46.20 SEEDbody eff−	131	83.9	5.50E−09	1.12E+05	6.16E−04
NKp46.21 SEEDbody eff−	92	95.7	1.94E−09	7.29E+05	1.41E−03
NKp46.22 SEEDbody eff−	164	95.5	5.34E−09	2.70E+05	1.44E−03
NKp46.23 SEEDbody eff−	80	98.5	2.20E−09	1.06E+05	2.34E−04
NKp46.24 SEEDbody eff−	19	95.3	5.58E−09	9.57E+04	5.34E−04
NKp46.25 SEEDbody eff−	72	95	2.12E−09	3.43E+05	0.000728
NKp46.26 SEEDbody eff−	146; 152^a^	95.4; 86.9^a^	1.46E−08	3.27E+05	4.80E−03
NKp46.27 SEEDbody eff−	198	95.5	6.85E−09	2.54E+05	1.74E−03
NKp46.28 SEEDbody eff−	102	94.7	2.85E−09	1.68E+05	4.78E−04
NKp46.29 SEEDbody eff−	150	81.8	<1.0E−12	1.05E+05	<1.0E−07
NKp46.31 SEEDbody eff−	168	94.4	2.55E−08	3.94E+04	1.00E−03
NKp46.34 SEEDbody eff−	121	96.3	1.93E−09	2.93E+05	5.66E−04

*Note*: Nonbinding molecules were excluded from this table. *k*_on_ is the rate constant of association, while *k*_off_ is the rate constant of dissociation.

^a^ Indicate the values for a second expression of the respective molecules.

Initial binding experiments utilizing BLI at a NKp46 concentration of 100 nM revealed specific antigen binding of 30 out of 40 VHH‐based NKCEs (Table 1). Consequently, these 30 NKp46‐specific SEEDbodies eff− were considered for further characterization. BLI was also exploited to analyze simultaneous binding to EGFR and NKp46. To this end, rhEGFR (ECD) was immobilized to the sensor tips, followed by a first association with the bispecific NKCE. Subsequently, a second association step was performed with the extracellular portion of rhNKp46 (Figure S1). This unveiled simultaneous binding on the protein level for all NKp46‐binding SEEDbodies eff−, whereas NKp46.37 SEEDbody eff−, which already did not show any binding to NKp46, also did not exhibit simultaneous binding behavior. Affinities with respect to NKp46 binding of VHH‐based NKCE ranged from the lower double digit nanomolar range (NKp46.16, NKp46.17, NKp46.26 and NKp46.31 SEEDbodies eff−) to binding in the sub‐nanomolar range (NKp46.5, NKp46.10, NKp46.29 SEEDbodies eff−) with most of the molecules displaying affinities in the single digit nanomolar range (Table 1).

### 2.2 NKp46‐specific VHH‐based NKCEs elicit NK cell‐mediated lysis of EGFR overexpressing tumor cells

**FIGURE 2:**
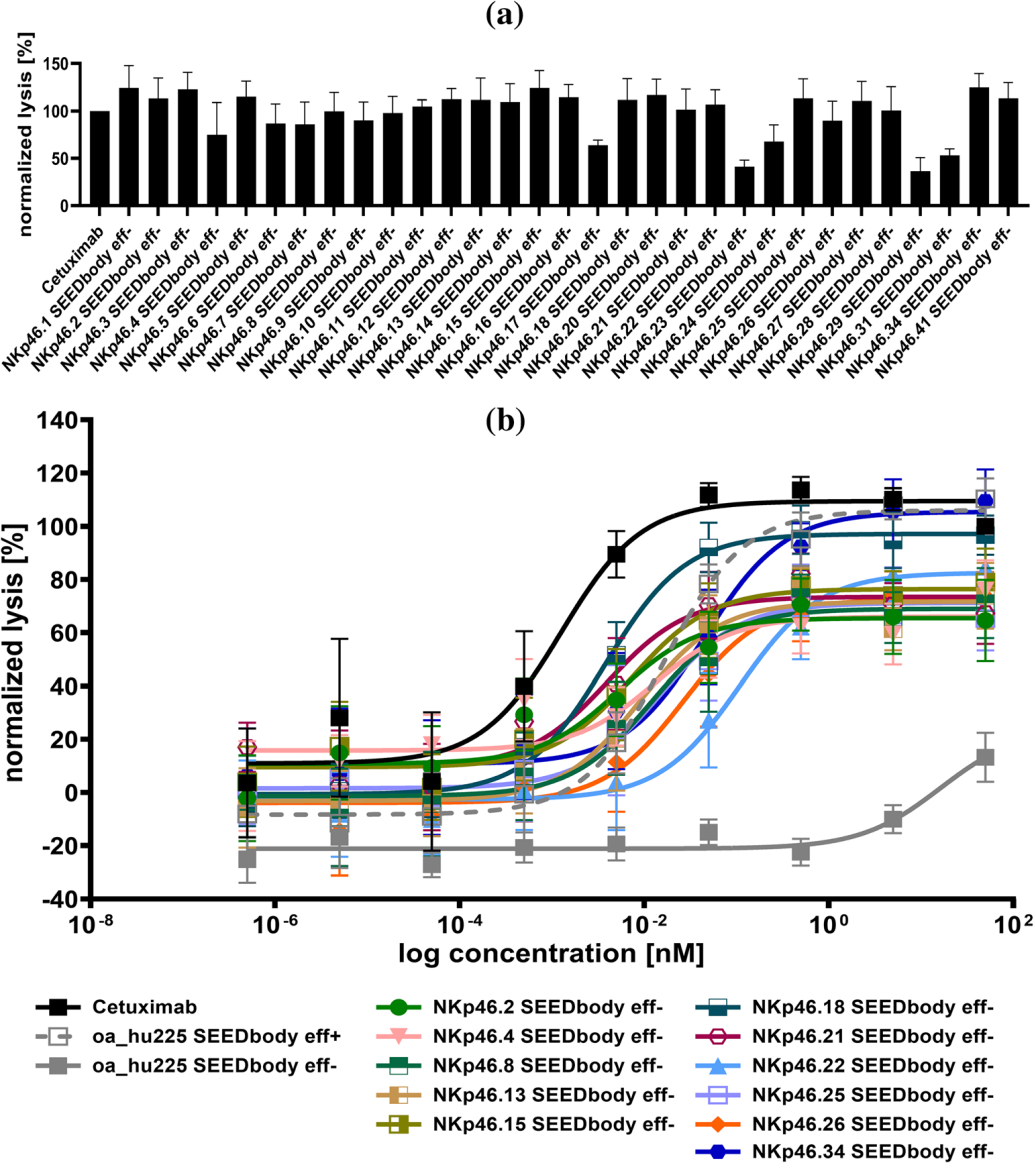
Fc‐silenced EGFR × NKp46 NKCEs trigger NK cell‐mediated lysis of EGFR‐overexpressing A431 cells. (a) Fluorescence based killing assays were conducted using A431 cells and freshly isolated PBMC‐derived NK cells derived from healthy donors at an effector‐to‐target cell (E:T) ratio of 5:1. Bispecific NKp46‐specific VHH SEEDbodies harboring a humanized version of the Fab arm of Cetuximab as well as an effector‐silenced Fc region were added at a concentration of 50 nM. As positive control, the monoclonal antibody Cetuximab, activating NK cells exclusively via FcγRIIIa was included. Mean values ± SEM of four independent experiments with biological duplicates are indicated. Data were normalized to the maximum concentration of Cetuximab to allow for comparison. (b) Fluorescence based killing assays of 11 selected NKCEs in a dose‐dependent manner were conducted with A431 cells and freshly isolated PBMC‐derived NK cells from healthy donors at E:T = 5:1. Cetuximab and a one‐armed effector competent SEEDbody lacking the NKp46 VHH domain (oa_hu225 SEEDbody eff+) as well as the corresponding effector‐silenced counterpart (oa_hu225 SEEDbody eff−) were included as controls. Mean values ± SEM of seven independent experiments with biological duplicates are indicated. Data were normalized to the maximum concentration of Cetuximab to allow for comparison.

Initial functional analyses for the generated VHH‐based NKCEs were conducted using the EGFR‐overexpressing tumor cell line A431 as well as NK cells derived from PBMCs of four healthy donors. Cetuximab was included as positive control, since it is known that this EGFR‐directed antibody triggers very potent NK cell‐mediated eradication of EGFR expressing tumor cells via ADCC (Derer et al., 2012, 2014). All compounds were assessed in terms of killing capacities at a concentration of 50 nM. Interestingly, while the extent of lysis differed noticeably between the studied molecules, all VHH‐based NKCEs significantly triggered NK cell‐dependent killing of A431 cells (Figure 2a). Importantly, killing of EGFR‐negative CHO cells was negligible, indicating tumor target‐specific redirection of NK cells by the herein generated NKp46 SEEDbodies eff− (Figure S2). Based on killing capacities, but also taking the sequence similarities of NKp46‐directed paratopes into account, 11 NKCEs were selected for a more meticulous characterization. To this end, killing of A431 cells was evaluated in a dose‐dependent manner using again PBMC‐isolated NK cells of healthy donors as effector cells (Figure 2b). As positive control, Cetuximab was again exploited, eliciting very potent NK cell‐mediated killing (EC_50_killing = 1.3 pM). For comparison we also utilized a monovalent (one armed) version of humanized Cetuximab, expressed as effector competent SEEDbody (oa_hu225 SEEDbody eff+). This molecule triggered lysis of A431 cells with a potency of 16.3 pM, whereas the same targeting arm in an effector‐silenced Fc backbone (oa_hu225 SEEDbody eff−) was not capable of significantly inducing NK cell‐dependent lysis of A431 cells. In contrast to this, all 11 selected VHH‐based NKp46‐specific NKCEs triggered dose‐dependent NK‐cell mediated lysis of A431 cells with potencies in the single digit picomolar to triple digit picomolar range. In this respect, NKp46.2 SEEDbody eff−, NKp46.18 SEEDbody eff−, and NKp46.21 SEEDbody eff− displayed the highest potencies with EC_50_killing values in the low single digit picomolar range, clearly demonstrating robust killing capacities mediated by camelid‐derived NKp46‐targeting sdAbs.

### 2.3 NKp46‐specific sdAb‐based NKCEs target distinct epitopes and mediate significant NK cell activation

**FIGURE 3:**
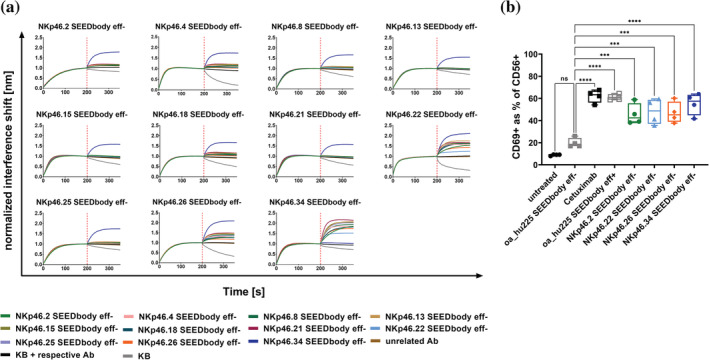
VHH‐based NKCEs target several distinct epitopes on NKp46 and trigger significant NK cell activation. (a) BLI sensograms showing competitive binding experiments of a panel of 11 NKp46 SEEDbodies for recombinant human NKp46 ECD. rhNKp46 ECD was immobilized to the sensor tip followed by a first association step using the respective SEEDbody at a concentration of 100 nM. Subsequently, a second association step was performed using another SEEDbody at 100 nM in the presence of 100 nM first analyte. In each experiment KB buffer as well as one SEEDbody in both association steps were included as controls. (b) Representation of CD69 expression for different NKCEs and control molecules within the CD56 positive NK cell population. Percentage of activation was determined by flow cytometric analysis via simultaneous NK cell staining with CD56 PE‐Cy7 and CD69 PE or respective isotype controls for appropriate gating adjustment (Figure S4). Graphs show box whiskers plots of four independent experiments measured in biological duplicates. ns, not significant; ***p < 0.001; ****p < 0.0001 versus oa_hu225 SEEDbody eff−.

To further characterize the NKCEs and determine epitope specificities, pairwise competition for binding to NKp46 was performed for all 11 NKp46 SEEDbodies eff− in every possible combination (Figure 3a and S3A). For this, BLI experiments were conducted in which NKp46 was immobilized to the sensor tip, followed by two consecutive association steps utilizing distinct NKp46 SEEDbodies eff−. This revealed two groups sharing nonoverlapping epitopes (epitope bin 1 for SEEDbodies eff− NKp46.2, NKp46.4, NKp46.8, NKp46.13, NKp46.15, NKp46.18, NKp46.21 and NKp46.25, and epitope bin 2 for NKp46.34 SEEDbody eff−). Interestingly, while consecutive binding was observed for NKp46.22 SEEDbody eff− and NKp46.34 SEEDbody eff− as well as NKp46.26 SEEDbody eff− and NKp46.34 SEEDbody eff−, successive binding was significantly diminished but still detectable for both clones and NKCEs that clustered to epitope bin 1. The same was true for competitive binding between both clones, NKp46.22 SEEDbody eff− and NKp46.26 SEEDbody eff−. Hence, it is tempting to speculate that clones within epitope bin 1, as well as SEEDbodies eff− NKp46.22 and NKp46.26 share overlapping, but distinct epitopes on NKp46 (Figure S3b).

Subsequently, we examined NK cell activation mediated by the herein described NKp46‐specific NKCEs in the presence of EGFR‐overexpressing A431 cells. To this end, upregulation of CD69 as early NK cell activation marker was analyzed for NKp46.2 SEEDbody eff− as representative clone of epitope bin 1 as well as NKp46.22 SEEDbody eff−, NKp46.26 SEEDbody eff− and NKp46.34 SEEDbody eff−, each targeting a unique epitope on NKp46 (Figure 3b and S4). All four NKCEs triggered significant activation of NK cells compared to the Fc effector‐silenced one‐armed EGFR‐targeting negative control (oa_hu225 SEEDbody eff−). Interestingly, NK cell activation was slightly lower for all NKCEs triggering NKp46 than for Cetuximab as well as compared with a monovalent Fc effector competent humanized version of Cetuximab, both mediating NK cell activation via FcγRIIIa ligation, clearly highlighting the impact of FcγRIIIa as very potent trigger molecule for NK cell activation.

### 2.4 Killing capacities of NKp46‐targeting NKCEs can be augmented by co‐engagement of FcγRIIIa

**FIGURE 4:**
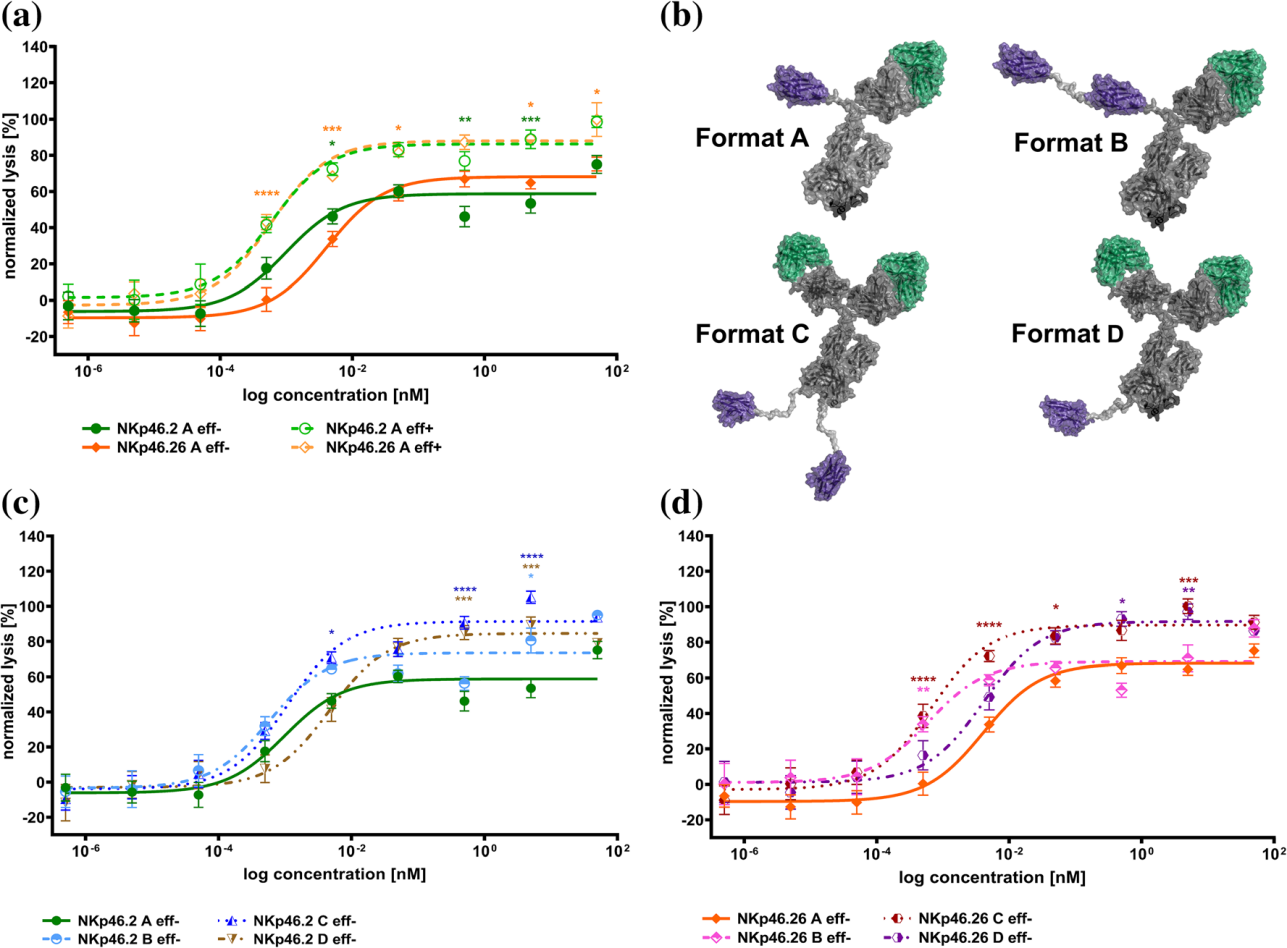
Format and Fc engineering approaches augment killing capacities of VHH‐based NKp46 × EGFR NKCEs. (a) Fluorescence based killing assays were conducted with EGFR‐overexpressing A431 cells and freshly isolated PBMC‐derived NK cells from healthy donors at an E:T ratio of 5:1 with increasing concentrations of strictly monovalent NKp46 and EGFR targeting NKp46.2 (green) and NKp46.26 (orange) VHH SEEDbodies with effector‐silenced (indicated as eff−, continuous lines and filled symbols) or effector competent (indicated as eff+, dotted lines and open symbols) Fc portions. (b) Schematic depiction of engineered antibody architectures for NK redirection based on a NKp46 specific VHH molecule in combination with humanized Cetuximab Fab in an effector silenced Fc backbone. Strictly monovalent *N‐*terminal fusion of NKp46 VHH and EGFR Fab shown as SEEDbody format A, while *N‐*terminal bivalent tandem arrangement of NKp46‐specific VHHs and monovalent EGFR Fab is indicated as SEEDbody format B. *N‐*terminal bivalent EGFR Fab fusion with *C*‐terminal bivalent arrangement of NKp46 VHH fused onto an IgG1 backbone is indicated as design C and monovalent *C‐*terminal NKp46 VHH fusion with bivalent *N‐*terminal EGFR Fab orientation is indicated as design format D. Schemes were generated using PyMol software version 2.3.0. (c) Fluorescence based killing assays with NKp46.2 based NKCE formats were conducted with EGFR‐overexpressing A431 cells and freshly isolated PBMC‐derived NK cells from healthy donors at an E:T ratio of 5:1 with increasing concentrations of NKp46.2 design A (green continuous line and filled symbols), design B (light blue, dotted line and open symbols), design C (blue, dotted line and open symbols) as well as design D (brown, dotted line and open symbols). (d) Fluorescence based killing assays with NKp46.26 based NKCE formats were conducted with EGFR‐overexpressing A431 cells and freshly isolated PBMC‐derived NK cells from healthy donors at an E:T ratio of 5:1 with increasing concentrations of NKp46.26 design A (orange continuous line and filled symbols), design B (pink, dotted line and open symbols), design C (dark red, dotted line and open symbols) as well as design D (purple, dotted line and open symbols). For all experiments, mean values ± SEM of eight independent experiments with biological duplicates are indicated. Data were normalized to the maximum concentration of Cetuximab to allow for comparison. *p < 0.05; **p < 0.01; ***p < 0.001; ****p < 0.0001 versus respective strictly monovalent bispecific SEEDbody eff− (design A).

Our group recently described that killing capacities of NKp30‐directed EGFR‐targeting bispecific NKCEs can be further enhanced by co‐triggering FcγRIIIa (Klausz et al., 2022; Pekar et al., 2021). This was also demonstrated by Vivier and colleagues for NKp46‐specific Fab‐derived paratopes incorporated into multifunctional NKCEs (Gauthier et al., 2019). To investigate, if this also holds true for sdAb‐derived NKCEs specific for NKp46, SEEDbodies NKp46.2 and NKp46.26 were expressed harboring an effector function enabled Fc region (eff+). These two sdAb‐based paratopes were chosen because of differences in affinities, epitope targeting and initial killing capacities (Table 1, Figures 2, 3a and S3). Intriguingly, co‐engagement of FcγRIIIa augmented both, potencies as well as efficacies (maximal lysis) for each studied NKCE (Figure 4a). In this respect, for NKp46.2 SEEDbody we observed a moderate enhancement in EC_50_killing (EC_50_ NKp46.2 SEEDbody eff− of 0.98 pM vs. EC_50_ NKp46.2 SEEDbody eff+ of 0.61 pM). This effect was even more pronounced for NKp46.26 SEEDbody, where potencies were augmented by a factor of seven (EC_50_ NKp46.26 SEEDbody eff− of 4.1 pM vs. EC_50_ NKp46.26 SEEDbody eff+ of 0.59 pM), corroborating the impact of the Fc backbone for NKCEs targeting NCRs.

### 2.5 Antibody format engineering enables the generation of NKp46‐specific VHH‐based NKCEs with enhanced potencies and efficacies

In a further attempt to improve killing capacities, we set out to investigate the influence of valencies for each paratope on tumor cell lysis as well as the impact of the overall antibody design architecture (Figure 4b). To this end, three additional formats were employed, all harboring an effector‐silenced Fc region (Figure 4b, design architecture B–D) as well as either VHHs NKp46.2 or NKp46.26. While initially, all molecules were tested harboring strictly monovalent *N‐*terminally linked paratopes (Figure 4b, format A), we also produced molecules allowing for bivalent targeting of NKp46. For this, the respective VHHs were engrafted in a tandem arrangement separated by a 20 amino acid Gly/Ser linker and fused to the *N*‐terminus of the AG chain of the SEED, while on the GA chain the Fab arm of hu225 was utilized (format B). In addition, we fused the respective VHH to the *C‐*terminus of an effector‐silenced IgG1 derivative of hu225, enabling bivalency for both paratopes (design C; of note a 20 amino acid Gly/Ser linker was implemented between the Fc part and the VHH). Finally, fusion of the Fab arm of hu225 to both *N‐*termini of the SEED chain as well as engraftment of only one respective VHH to the *C‐*terminus of the AG chain of the SEED (separated by a 20 amino acid Gly/Ser linker) enabled bivalent targeting of EGFR, as well as addressing Nkp46 in a monovalent fashion (format D). In general, all different formats could successfully be produced in Expi293 cells with expression yields in the double to triple digit milligram per liter scale (Table 2). Interestingly, slightly reduced expression yields were observed for both NKp46‐specific sdAbs when engrafted in asymmetric design architecture D (double digit mg/L vs. triple digit mg/L), suggesting an overall reduced productivity for this particular format. Besides, molecules representing different antibody architectures unveiled a high purity after expression and affinity purification. Except for NKp46‐specific VHH NKp46.26 in format A (showing a slightly broader target peak with 86.9% purity), size exclusion chromatography (SEC) profiles showed target monomer peak purities of above 90% (Table 2, Figure S5). Also, thermal stabilities were quite similar between the different formats for a given engrafted VHH paratope, that is, Tm1 ranging from 64.1 to 68.5°C for NKp46.2 and 54.9 to 56.0°C for NKp46.26, with VHH NKp46.2 in format C (IgG1‐based format) that seemed to be the most stable design exhibiting its first unfolding transition midpoint at 68.5°C. Additionally, a lower overall thermostability for all NKp46.26 harboring molecules was found (Table 2, Figure S6). Furthermore, we employed HIC analysis in order to determine the relative hydrophobicity of the generated NKCE architectures (Table 2, Figure S7). Therefore, we used two therapeutic antibodies, Cetuximab and Avelumab (HIC retention times of 5.8 and 7.2 min, respectively), that were granted marketing approval by the FDA as reference. In general, we observed a trend toward higher retention times and hence, for higher hydrophobicity, when more paratopes were incorporated into a given molecule and for the designs where the NKp46 VHHs were fused to the *C*‐terminus of the molecule (design C and D). In this respect, HIC retention times were 6.8 and 6.5 min, respectively, for molecules NKp46.2 C and NKp46.26 C that harbor four paratopes in total. In contrast to this, HIC retention times were 6.1 and 5.8 min when both paratopes were engrafted into the strictly monovalent format A. Consequently, the NKCE architectures incorporating three paratopes, that is, design B and D, showed intermediary HIC retention times with NKp46.2 B and NKp46.26 B eluting after 6.3 and 5.8 min as well as NKp46.2 D and NKp46.26 D eluting after 6.6 and 6.5 min, respectively. Overall, HIC retention times of all different molecules were between those of Cetuximab and Avelumab, clearly demonstrating adequate biophysical properties for herein engineered NKCE architectures.

**TABLE 2:** Biophysical and functional properties of different VHH‐based EGFR × NKp46 NKCE format architectures.

#	Yield (mg/L)	SEC (%)	Mean Tm 1 (°C)	HIC retention time (min)	EC_50_killing (pM)
NKp46.2 A eff−	176	92.1	64.5	6.1	0.98
NKp46.26 A eff−	152	86.9	55.4	5.8	4.1
NKp46.2 B eff−	222	91.7	64.1	6.3	0.59
NKp46.26 B eff−	120	94.4	54.9	5.8	0.6
NKp46.2 C eff−	120	94.2	68.5	6.8	1.08
NKp46.26 C eff−	116	96.3	55.6	6.5	0.67
NKp46.2 D eff−	85	93.5	65.5	6.6	4.67
NKp46.26 D eff−	52	98.1	56.0	6.5	4.11

Finally, we set out to investigate the ability of the different NKCE formats to redirect NK cell cytotoxicity against EGFR‐overexpressing A431 cells. Similar to the initially observed results in format A (Figure 2b), both NKp46‐directed VHHs triggered lysis in the low picomolar range (EC_50_ NKp46.2 of 0.98 pM, EC_50_ NKp46.26 of 4.1 pM; Figure 4a,c,d). Bivalent targeting of activating receptor NKp46 on the NK cell in format B augmented killing capacities for both engrafted VHHs. Intriguingly, for VHH NKp46.2 we observed not only a slightly enhanced potency in format B (EC_50_ NKp46.2 B of 0.59 pM vs. EC_50_ NKp46.2 A of 0.98 pM) but also a trend toward higher efficacies, that is, maximum lysis (Figure 4c). For NKp46.26 the effect was even more distinct in terms of potencies (EC_50_ NKp46.26 B of 0.60 pM vs. EC_50_ NKp46.26 A of 4.1 pM), resulting in an improvement of approximately sevenfold. However, bivalent targeting of NKp46 had no impact on maximum killing capacities (Figure 4d). This is in strong contrast to the design architectures that allow for bivalent targeting of EGFR (Figure 4c,d, format C and D). Here, maximum killing capacities were significantly enhanced compared to both formats that only enable monovalent targeting of the tumor associated antigen. Though, for VHH NKp46.2 no significant benefit was observed in terms of potencies (EC_50_ NKp46.2 C of 1.08 pM vs. EC_50_ NKp46.2 A of 0.98 pM). In fact, potencies seem to be even reduced when reformatted into format D, allowing for bivalent targeting of EGFR and monovalency for NKp46 (EC_50_ NKp46.2 D of 4.67 pM vs. EC_50_ NKp46.2 A of 0.98 pM. Yet, this is partially misleading, since significantly enhanced efficacies result in limited comparability). Opposed to these findings, for VHH NKp46.26 bivalent targeting of EGFR and for NKp46 resulted in both, enhanced potencies by the factor of approximately 6‐fold (EC_50_ NKp46.26 C of 0.67 pM vs. EC_50_ NKp46.26 A of 4.1 pM) as well as significantly augmented maximal killing capacities (Figure 4d). Even in design D reformatting of this particular sdAb largely maintained potencies albeit displaying much higher efficacies. Importantly, for all molecules analyzed killing of EGFR‐negative CHO cells was negligible (Figure S8). Ultimately, this shows that killing capacities of VHH‐derived NKp46‐specific NKCEs can be significantly augmented by antibody format engineering. Of note, our data suggests that molecular attributes such as the targeted epitope or the spatial orientation of individual paratopes within the molecular architecture may affect the cytotoxic potential in either a positive or negative way.

## 3 DISCUSSION

In this work, we have generated potent NKCE formats that bridge NKp46 on NK cells with EGFR on tumor cells. NKp46 is an activating receptor expressed on NK cells, belonging to the group of NCRs. It was previously shown by Vivier and co‐workers that NKp46 can be effectively targeted for the generation of efficacious NKCEs and currently, this approach is being explored in clinical development (e.g., NCT05086315) (Gauthier et al., 2019). The authors generated NKp46‐directed paratopes based on canonical VH as well as VL comprising antigen binding sites and furthermore characterized several different sophisticated NKCE formats. Our group has recently described an efficient route for generating NKCEs based on NKp30‐directed camelid‐derived sdAbs (Klausz et al., 2022). This strategy involved immunization of camelids followed by YSD‐based antibody selection. sdAbs such as camelid‐derived VHH domains afford the benefit of multiple reformatting options owing to their simple structure and composition compared to canonical paratopes (Könning et al., 2017; Krah et al., 2016; Pekar et al., 2020; Yanakieva et al., 2022). Moreover, sdAbs can be readily obtained using different display technologies (Pardon et al., 2014; Roth et al., 2020; Sellmann et al., 2020; Valldorf et al., 2022). We applied this strategy in this study for the generation of NKp46‐based NKCEs. Following camelid immunization and YSD, we were able to isolate a diverse panel of sdAb paratopes that were subsequently reformatted into NKCEs enabling targeting of both, EGFR and NKp46, in a monovalent fashion (Figure 4b, design A). Characterization of a panel of NKCEs revealed dose‐dependent triggering of NK cell mediated lysis of EGFR‐overexpressing tumor cells with potencies in the picomolar range. Similar potencies were previously observed by our group for equivalent NKCEs harboring NKp30‐specific VHH domains (Klausz et al., 2022) as well as recapitulated in this study (Figure S9). NKp30 is another activating receptor on NK cells belonging to the group of NCRs (Pende et al., 1999). In contrast to NKp46, the expression of NKp30 on tumor infiltrating NK cells is downregulated while it is more consistently expressed on NK cells in the blood of cancer patients (Demaria et al., 2022). Moreover, NKp30 is also displayed by other immune cell subsets that might be beneficial in therapeutic settings (Correia et al., 2018; Hudspeth et al., 2012). Ultimately, expression profiles of the respective NCR as well as its distribution on other immune cells need to be taken into account when designing NKCEs for a given indication.

Additionally, we set out to augment the cytotoxic potential of NKp46‐based NKCEs by format and Fc engineering. For this, we focused on VHHs that address distinct epitopes and elicit a robust activation of NK cells. Similar to what has been shown by Vivier for NKp46 (Gauthier et al., 2019) and our group for NKp30 (Klausz et al., 2022; Pekar et al., 2021), co‐engagement of FcγRIIIa by utilizing an effector functional Fc portion enhanced killing capacities of VHH‐based NKCEs.

Intriguingly, we were able to significantly improve potencies and efficacies of NKp46‐directed NKCEs by format engineering. Bivalent targeting of NKp46 was beneficial in enhancing potencies for both VHHs, while bivalency for EGFR significantly improved maximum killing. Consequently, bivalent targeting of both, the TAA and the trigger molecule on NK cells enabled strongest augmentation of NK cell mediated lysis capacities, while unspecific lysis of target‐negative cells was negligible. Noteworthy, the magnitude of improvement differed between both VHHs which target different epitopes on NKp46. It was previously shown by Chaparro‐Riggers and colleagues that redirection capacities of T cell engagers highly depend on the epitope location as well as on the overall geometry of the engager molecule (Chen et al., 2021). The herein presented investigations are supporting the notion that the epitope on the effector cell trigger receptor as well as the valencies and the spatial orientation of the individual paratopes within the molecular architecture are important factors impacting killing capacities that ultimately need to be considered when designing NKCEs.

## 4 MATERIALS AND METHODS

### 4.1 Camelid immunization

All procedures and animal care were in accordance with local animal welfare protection laws and regulation. Of note, all procedures involving animals were conducted at preclinics GmbH, Germany. Animals remained alive after immunization and final blood collection. For the immunization, two Alpacas (*Vicugna pacos*) and one Llama (*Lama glama*) were immunized with recombinant human (rh) NKp46 extracellular domain (ECD; Acro Biosystems). The immunization protocol comprised four administrations of 300 μg rh NKp46 ECD, each conducted as subcutaneous injections at three sites, over a period of 42 days in total (at d0, d14, d28 and d35). For this, the antigen was diluted to a stock concentration of 1 mg/mL in PBS and emulsified either with Complete Freund's Adjuvant, for initial immunization, or with Incomplete Freund's Adjuvant for subsequent immunizations. Seven days after the final administration (d42), a volume of 100 mL blood per specimen was collected prior to RNA extraction and subsequent cDNA synthesis.

### 4.2 Yeast strains and media

*Saccharomyces cerevisiae* strain EBY100 (*MATa URA3‐52 trp1 leu2Δ1 his3Δ200 pep4::HIS3 prb1Δ1.6R can1 GAL (pIU211:URA3*)) (Thermo Fisher Scientific) was employed for yeast surface display. Cells were cultivated in yeast extract–peptone–dextrose (YPD) medium composed of 20 g/L peptone, 20 g/L dextrose and 10 g/L yeast extract supplemented with 10 mg/mL penicillin–streptomycin (Gibco). After homologous recombination‐based cloning, cells harboring library plasmids (pDisp) were cultivated in medium using minimal synthetic defined (SD)‐base (Takara Bio) and corresponding dropout mix (Takara Bio) composed of all essential amino acids except for tryptophan (−Trp) for selection, supplemented with 5.4 g/L Na_2_HPO_4_ and 8.6 g/L NaH_2_PO_4_ ∙ H_2_O. To induce antibody gene expression, cells were transferred into galactose containing SG dropout medium (−Trp), consisting of SG‐base medium (Takara Bio) supplemented with 10% (w/v) polyethylene glycol 8000 (PEG 8000).

### 4.3 Plasmids for yeast surface display and library generation

Gap repair cloning based on homologous recombination in yeast was exploited for the generation of VHH libraries. Protocols for PCR amplification of VHH fragments as well as library construction have already been described by our group (Roth et al., 2020). In brief, digestion of the display plasmid pDisp with specific restriction enzyme *Bsa*I followed by genetic fusion of VHH library candidates in frame to Aga2p by replacement of a stuffer sequence due to gap repair cloning allowed for the presentation of sdAb variants on the yeast cell surface. The additional insertion of a HA epitope linked *C*‐terminally to Aga2p on the pDisp backbone enabled the detection of proper full‐length VHH presentation on the yeast surface.

### 4.4 Library sorting

For sorting purposes, EBY100 cells were grown overnight in SD medium with dropout mix lacking tryptophan (−Trp) at 30°C and 120 rpm prior to induction of surface expression by cell transfer into SG medium with dropout mix (−Trp) at 10^7^ cells/mL and 48 h incubation at 20°C. Antigen binding was detected by indirect immunofluorescence using 1 μM rh his‐tagged NKp46 ECD (Acro Biosystems) in combination with anti‐his mouse monoclonal detection antibody (SureLight® Allophycocyanin, Abcam, diluted 1:20). Simultaneous monitoring of full‐length VHH surface expression by application of a FITC‐labeled rabbit polyclonal anti‐HA antibody (Abcam, diluted 1:20) allowed for a two‐dimensional sorting strategy (Figure 1b). The fluorescence activated cell sorting (FACS) procedure was performed on a BD FACSAria™ Fusion cell sorter (BD Biosciences) device. Control samples, that is, cells incubated with secondary labeling reagents only or cells incubated with secondary labeling reagents and his‐tagged NKp46 or unrelated antigen as well as untreated cells were employed in every experiment, allowing for gate adjustment of the desired cell population.

### 4.5 Protein expression and purification

After sequencing of FACS enriched populations and subsequent clone selection, the VHH variants were fused *N‐*terminally to the hinge region of Fc immune effector‐silenced (eff−) SEED AG chains prior to cloning into pTT5 mammalian expression vector (Durocher, 2002), ultimately enabling the production of eff− bispecific SEEDbodies (SEEDbody eff−) in combination with eff− humanized Cetuximab Fab on the SEED GA chain for the initial protein characterization. For a more detailed characterization, specific VHHs were also expressed as effector competent SEEDbodies (SEEDbody eff+) and in different orientations and valencies (as eff− versions). For protein expression, Expi293 cells were transiently transfected with respective pTT5 vectors according to the manufacturer's instructions (Thermo Fisher Scientific). The protein containing supernatants were harvested 6 days post transfection by centrifugation and purified via MabSelect antibody purification chromatography resin (GE Healthcare), followed by a buffer exchange step to PBS pH 6.8 overnight using Pur‐A‐Lyzer™ Maxi 3500 Dialysis Kit (Sigma Aldrich). Resulting molecule concentrations were measured using Nanodrop ND‐1000 (Peqlab) after sterile filtration with Ultrafree®‐CL GV 0.22 μm centrifugal devices (Merck Millipore).

### 4.6 Protein analytics

For the assessment of protein sample quality regarding target monomer peaks (%), analytical size exclusion chromatography (SEC) was conducted, using 7.5 μg protein per sample on a TSKgel UP‐SW3000 column (2 μm, 4.6 × 300 mm, Tosoh Bioscience) in an Agilent HPLC 1260 Infinity system with a flow rate of 0.35 mL/min using 50 mM sodium phosphate, 0.4 M NaClO_4_ pH 6.3 as mobile phase. Hydrophobicity of the different molecules was determined by hydrophobic interaction chromatography (HIC) using 20 μg protein per sample on a TSKgel Butyl‐NPR column (2.5 μm, 4.6 × 100 mm, Tosoh Bioscience) in an Agilent HPLC 1260 Infinity system with a flow rate of 0.5 mL/min. Samples were premixed with 50% (v/v) 2 M ammonium sulfate solution prior to injection. A gradient running from mobile phase A (1.2 M ammonium sulfate in PBS) to mobile phase B (50% methanol in 0.1× PBS) over 15.0 min at 25°C was applied. Signals were recorded at 214 nm. Anti‐PD‐L1 Avelumab and anti‐EGFR Cetuximab were used as reference molecules. Thermal unfolding of the antibodies was assessed by differential scanning fluorimetry (DSF) on a Prometheus NT.PLEX nanoDSF instrument. Samples were measured in duplicates using nanoDSF grade standard capillaries. A temperature gradient from 20 to 95°C at a slope of 1°C/min was used while recording fluorescence at 350 and 330 nm. Unfolding transition midpoints (Tm) were determined from the first derivative of the fluorescence ratio 350 nm/330 nm.

### 4.7 Molecular modeling and in silico property prediction

To create homology models of the full length IgGs and VHHs the antibody modeler tool in the molecular modeling software package moe (Mol Operating Enrion 2020.09: Chemical Computing Group Inc.; 2020) was utilized. The generation of IgG‐VHH constructs were built by adding linkers via moe's protein builder, followed by a conformational search of the linker via moe's linker modeler. Finally, an energy minimization was performed, treating the linker as flexible and the IgG and VHH domains as rigid bodies. Visualization of 3D structures was done with PyMOL (The PyMOL Molecular Graphics System, Version 2.0 Schrödinger, LLC.).

The in silico developability profile was computed using an internal pipeline termed “Sequence Assessment Using Multiple Optimization Parameters (SUMO)” (Evers et al., 2022).Briefly, this approach automatically generates VHH models based on the provided sequences, identifies the human‐likeness by sequence comparison to the most similar human germline sequence, determines structure‐based surface‐exposed chemical liability motifs (unpaired cysteines, methionines, asparagine deamidation motifs and aspartate deamidation sites) as well as sites susceptible to post‐translational modification (*N*‐linked glycosylation). Moreover, a small set of orthogonal computed physico‐chemical descriptors including the isoelectric point (p*I*) of the variable domain, Schrodingers AggScore as predictor for hydrophobicity and aggregation tendency calculated for the complete variable domain as well as the complementarity‐determining regions (CDRs) only and the calculated positive patch energy of the CDRs were determined (Sankar et al., 2018). These scores were complemented with a green to yellow to red color coding, indicating scores within one standard deviation from the mean over a benchmarking dataset of multiple biotherapeutics approved for human application as green, scores above one standard deviation as yellow and those above two standard deviations as red (Ahmed et al., 2021) (Table S1). For the AggScore values, these cutoffs were slightly adjusted based on correlation analyses to internal experimental HIC data.

### 4.8 Biolayer interferometry

For binding assays with recombinant proteins, the Octet RED96 system (ForteBio, Pall Life Science) was employed using 25°C and 1000 rpm agitation settings. In order to determine binding kinetics, bispecific molecules were loaded on anti‐human Fc (AHC) Biosensors at 3 μg/mL in PBS for 3 min followed by 60 s sensor rinsing in kinetics buffer (KB; PBS + 0.1% Tween‐20 and 1% bovine serum albumin, BSA). Afterwards, binding to human NKp46 ECD (Acro Biosystems) in decreasing concentrations ranging from 100 to 1.56 nM in KB was measured for 300 s followed by dissociation for 300 s in KB. In each experiment, one negative control using irrelevant antigen and a second reference by incubating the antibody in KB instead of the antigen was measured.

Simultaneous binding capacities of NKCEs were measured by loading his‐tagged EGFR ECD (produced in‐house) on anti‐his tips (HIS1K) for 3 min at 3 μg/mL in PBS. After sensor rinsing a first binding step was performed using the respective NKCE at 100 nM, followed by a consecutive association step with 100 nM of NKp46 Fc‐fusion protein (Acro Biosystems). Parallel control measurements for each association step of Biosensors incubated in KB instead were utilized.

To analyze competitive binding of VHHs, his‐tagged NKp46 ECD was loaded at 3 μg/mL in PBS for 3 min to HIS1K Biosensors followed by 60 s sensor rinsing in KB. Association of the bispecific antibodies (100 nM) for 200 s in KB was followed by an additional association step with a different SEEDbody for another 150 s in KB in presence of 100 nM first analyte. Control values using an unrelated antibody or the same bispecific SEEDbody for both association steps as well as controls using KB buffer were included.

Data were fitted and analyzed with ForteBio data analysis software 8.0 using a 1:1 binding model after Savitzky–Golay filtering.

### 4.9 Tumor cell killing assays

A detailed protocol has previously been described by our group and can be found elsewhere (Pekar et al., 2020). In brief, peripheral blood mononuclear cells (PBMCs) were freshly isolated from healthy donors. Subsequently, NK cells were enriched using EasySep™ Human NK Cell Isolation Kit (Stemcell Technologies). Purified NK cells were rested overnight in complete medium supplemented with low dose rh IL‐2 (100 U/mL, R&D systems) prior to cell adjustment to 0.625 × 10^6^ viable cells/mL the next day. EGFR overexpressing A431 cells or EGFR negative ExpiCHO™ cells were prepared by cell staining with CellTracker™ Deep Red Dye (ThermoFisher) and seeded into a 384‐well clear bottom microtiter plate (Greiner Bio‐One) at 2500 cells/well. After a 3 h adherence period, NK effector cells were dispensed to target cells at a effector to target cell (E:T) ratio of 5:1 before addition of bsAbs at concentrations as indicated followed by incubation for 24 h in the Incucyte® system. As negative control, a monovalent EGFR targeting Fc effector‐silenced antibody derivative was used (oa_hu225 SEEDbody eff−). Dead cells were detected by dispensing 0.03 μM SYTOX™ Green Dead Cell Stain (Invitrogen) to the assay. Cell lysis was normalized to maximum lysis induced by Cetuximab or to target cells cultivated with 30 μM staurosporine (Merck Millipore). Overlay signals allowed for analysis of dead target cells only, while subtraction of overlay signals from overall green signal enabled specific analysis of dead NK cells.

### 4.10 NK cell activation assay

To determine specific NK cell activation by herein engineered bsAbs, 20,000 A431 cells/well were seeded in 96‐well V‐bottom microtiter plates (Thermo Fisher Scientific) and rested 3 h for adherence prior to the addition of 100,000 NK cells/well, resulting in and E:T ratio of 5:1. Antibodies were added at a final concentration of 50 nM followed by 24 h incubation at 37°C. For NK surface receptor staining, cells were washed once with PBS + 1% BSA, followed by incubation with anti‐CD69 PE (R&D Systems) and anti‐CD56 PE‐Cy7 (Beckman Coulter) or respective isotype controls for 1 h on ice. After another washing step, cells were analyzed by flow cytometry employing the IntelliCyt® iQue® Screener Plus system (Sartorius). For measurement and compensation of fluorochromes the IntelliCyt® ForeCyt® Enterprise Client Edition 8.0 (R3) Version 8.0.7430 software (Sartorius) was used. The applied gating strategy is shown in Figure S4.

### 4.11 Data processing and statistical analysis

Graphical and statistical analyses were conducted with GraphPad Prism 8 software. *P*‐values were calculated utilizing repeated measures ANOVA and the Bonferroni or Tukey post‐test as recommended, or the Student's *t*‐test when appropriate. *p* ≤ 0.05 were regarded as statistically significant.

## AUTHOR CONTRIBUTIONS

**Britta Lipinski:** Data curation (equal); formal analysis (equal); investigation (equal); methodology (lead); writing – original draft (equal). **Paul Arras:** Conceptualization (equal); data curation (equal); formal analysis (equal); investigation (equal); methodology (lead); resources (equal); software (equal). **Lukas Pekar:** Conceptualization (equal); formal analysis (equal); investigation (equal); methodology (lead); validation (equal). **Daniel Klewinghaus:** Investigation (supporting); methodology (equal); visualization (supporting). **Ammelie Svea Boje:** Methodology (supporting); software (supporting); visualization (supporting); writing – review and editing (supporting). **Simon Krah:** Formal analysis (equal); methodology (equal); resources (equal). **Jasmin Zimmermann:** Data curation (supporting); formal analysis (supporting); investigation (supporting); methodology (supporting). **Katja Klausz:** Conceptualization (supporting); formal analysis (supporting); investigation (supporting); visualization (supporting); writing – review and editing (supporting). **Matthias Peipp:** Data curation (supporting); supervision (equal); validation (supporting); visualization (supporting); writing – original draft (supporting). **Vanessa Siegmund:** Investigation (equal); methodology (equal); resources (supporting); software (supporting); validation (equal); visualization (supporting); writing – original draft (supporting); writing – review and editing (supporting). **Andreas Evers:** Data curation (equal); formal analysis (equal); investigation (supporting); methodology (supporting); resources (supporting); software (supporting); validation (supporting); visualization (equal); writing – original draft (supporting). **Stefan Zielonka:** Conceptualization (lead); investigation (lead); project administration (lead); supervision (lead); writing – original draft (lead); writing – review and editing (lead).

## CONFLICT OF INTEREST STATEMENT

LP, BL, PA, DK and SZ filed a patent application based on this work. In addition, LP, BL, PA, SK, JZ, AE, VS, DK and SZ are employees at Merck Healthcare KGaA. Besides, this work was conducted in the absence of any further commercial interest.

## Supporting information


**Appendix S1.** Supporting Information

## Data Availability

The data that supports the findings of this study are available in the supplementary material of this article.
