# Supplementary material for: NKp46‐specific single domain antibodies enable facile engineering of various potent NK cell engager formats
Source: Protein Sci. 2023 Feb 24;32(3):e4593. doi: 10.1002/pro.4593 (PMC9951198; doi:10.1002/pro.4593)
Supplement: Supplementary file 1 — Appendix S1. Supporting Information [file PRO-32-e4593-s001.docx]

**Supplementary Information: NKp46-specific single domain antibodies enable facile engineering of various potent NK cell engager formats**

**Running title: NK cell engagers based on NKp46-specific VHH domains**

Britta Lipinski^1,2+^, Paul Arras^1+^, Lukas Pekar^1+^, Daniel Klewinghaus^1^, Ammelie Svea Boje^3^, Simon Krah^1^, Jasmin Zimmermann^1,2^, Katja Klausz^3^, Matthias Peipp^3^, Vanessa Siegmund^4^, Andreas Evers^5^, and Stefan Zielonka^1,2*^

^1^Protein Engineering and Antibody Technologies, Merck Healthcare KGaA, Frankfurter Straße 250, D-64293 Darmstadt, Germany

^2^Institute for Organic Chemistry and Biochemistry, Technical University of Darmstadt, Alarich-Weiss Straße 4, D-64287 Darmstadt

^3^Division of Antibody-Based Immunotherapy, Department of Internal Medicine II, University Hospital Schleswig-Holstein and Christian-Albrechts-University Kiel, Rosalind-Franklin-Straße 12, D-24105 Kiel, Germany

^4^Protein and Cell Sciences, Merck Healthcare KGaA, Frankfurter Straße 250, D-64293 Darmstadt, Germany

^5^ Computational Chemistry and Biology, Merck Healthcare KGaA, Frankfurter Straße 250, D-64293 Darmstadt, Germany

**Table SI: *In silico* sequence assessment of enriched VHH domains exhibit overall favorable computed developability properties.**

| **ID** | **VARIABLE HEAVY** | | | **CDR LIABILITIES 3D** | | | | **CDR PTMs** | **IN SILICO PHYSCHEM** | | | |
| --- | --- | --- | --- | --- | --- | --- | --- | --- | --- | --- | --- | --- |
|  | MOST SIMILAR GERMLINE | SEQ-ID | SEQ-ID FW | NON-  CANONICAL CYS | MET OXIDATION | DEAMI-  DATION | ISO-  MERIZATION | N-  GLYCO-SYLATION | plFv 3D | Agg-Score | CDR Agg-Score | CDR Positive Patch Energy |
| NKp46.1 | IGHV3-23*01 | 80.5 | 81.3 | 0 | 0 | 0 | 1 | 0 | 8.5 | 97.9 | 63.1 | 518.2 |
| NKp46.2 | IGHV3-23*01 | 79.6 | 82.5 | 0 | 0 | 2 | 1 | 0 | 8.6 | 59.2 | 37.4 | 509.3 |
| NKp46.3 | IGHV3-23*01 | 77.1 | 83.8 | 0 | 0 | 1 | 1 | 0 | 8.8 | 78.6 | 50.2 | 480.7 |
| NKp46.4 | IGHV3-23*01 | 76.5 | 82.5 | 0 | 0 | 0 | 1 | 0 | 6.9 | 78.2 | 48.7 | 457.6 |
| NKp46.5 | IGHV3-23*01 | 77.4 | 82.5 | 0 | 0 | 0 | 1 | 0 | 7.6 | 74.4 | 41.8 | 504.9 |
| NKp46.6 | IGHV3-23*01 | 73.7 | 78.8 | 0 | 0 | 1 | 1 | 1 | 9.4 | 75.4 | 40.5 | 654.6 |
| NKp46.7 | IGHV3-23*01 | 73.7 | 77.5 | 0 | 0 | 1 | 2 | 1 | 8.5 | 65.0 | 29.9 | 551.7 |
| NKp46.8 | IGHV3-23*01 | 72.9 | 80.0 | 0 | 0 | 1 | 1 | 1 | 9.0 | 84.7 | 53.6 | 537.6 |
| NKp46.9 | IGHV3-23*01 | 72.9 | 78.8 | 0 | 0 | 1 | 0 | 0 | 8.5 | 48.5 | 21.8 | 501.7 |
| NKp46.10 | IGHV3-23*01 | 70.3 | 77.5 | 0 | 0 | 1 | 1 | 0 | 7.6 | 52.1 | 11.0 | 437.8 |
| NKp46.11 | IGHV3-23*01 | 76.1 | 81.3 | 0 | 1 | 2 | 0 | 0 | 8.9 | 93.3 | 84.8 | 649.6 |
| NKp46.12 | IGHV3-23*01 | 75.7 | 81.3 | 0 | 0 | 1 | 1 | 0 | 9.1 | 115.5 | 71.4 | 729.2 |
| NKp46.13 | IGHV3-23*01 | 75.7 | 81.3 | 0 | 0 | 0 | 2 | 0 | 7.8 | 75.0 | 54.0 | 485.5 |
| NKp46.14 | IGHV3-23*01 | 72.9 | 81.3 | 0 | 0 | 1 | 0 | 0 | 8.5 | 108.5 | 91.1 | 455.7 |
| NKp46.15 | IGHV3-23*01 | 77.0 | 82.5 | 0 | 0 | 1 | 0 | 0 | 8.5 | 74.2 | 57.1 | 425.3 |
| NKp46.16 | IGHV3-23*01 | 78.3 | 81.3 | 0 | 1 | 0 | 1 | 0 | 7.9 | 139.7 | 121.4 | 228.3 |
| NKp46.17 | IGHV3-23*01 | 77.9 | 82.5 | 0 | 1 | 1 | 1 | 0 | 8.1 | 40.7 | 34.0 | 487.4 |
| NKp46.18 | IGHV3-23*01 | 77.4 | 81.3 | 0 | 0 | 1 | 2 | 0 | 8.0 | 168.6 | 107.2 | 506.9 |
| NKp46.19 | IGHV3-23*01 | 72.9 | 78.8 | 0 | 0 | 0 | 1 | 0 | 5.8 | 108.4 | 76.0 | 380.9 |
| NKp46.20 | IGHV3-23*01 | 75.7 | 83.8 | 0 | 0 | 1 | 0 | 1 | 4.7 | 172.9 | 135.3 | 289.6 |
| NKp46.21 | IGHV3-23*02 | 71.7 | 77.5 | 0 | 0 | 0 | 2 | 0 | 7.9 | 53.3 | 31.4 | 663.2 |
| NKp46.22 | IGHV3-23*01 | 71.2 | 82.5 | 0 | 0 | 1 | 0 | 0 | 7.4 | 40.5 | 21.4 | 707.3 |
| NKp46.23 | IGHV3-23*02 | 71.1 | 76.3 | 0 | 0 | 0 | 2 | 0 | 6.0 | 111.5 | 61.4 | 358.8 |
| NKp46.24 | IGHV3-23*02 | 70.8 | 73.8 | 0 | 0 | 0 | 2 | 0 | 4.8 | 84.4 | 63.3 | 317.9 |
| NKp46.25 | IGHV3-23*01 | 72.0 | 75.0 | 0 | 0 | 0 | 1 | 1 | 7.7 | 165.2 | 133.7 | 540.7 |
| NKp46.26 | IGHV3-64*04 | 72.3 | 78.5 | 0 | 0 | 2 | 0 | 0 | 4.9 | 66.9 | 37.1 | 473.5 |
| NKp46.27 | IGHV3-48*01 | 73.7 | 82.5 | 0 | 1 | 2 | 2 | 0 | 7.6 | 128.8 | 86.8 | 408.2 |
| NKp46.28 | IGHV3-23*01 | 71.9 | 75.0 | 0 | 0 | 2 | 1 | 0 | 8.5 | 121.2 | 91.7 | 504.7 |
| NKp46.29 | IGHV3-23*01 | 69.3 | 73.8 | 0 | 0 | 0 | 2 | 0 | 7.9 | 152.2 | 97.2 | 343.8 |
| NKp46.30 | IGHV3-43*02 | 70.2 | 72.5 | 2 | 0 | 0 | 6 | 0 | 3.2 | 68.8 | 16.7 | 78.7 |
| NKp46.31 | IGHV3-23*05 | 69.9 | 73.8 | 0 | 0 | 2 | 1 | 0 | 7.9 | 85.2 | 50.6 | 430.9 |
| NKp46.32 | IGHV3-21*01 | 69.0 | 76.3 | 0 | 1 | 0 | 3 | 0 | 5.6 | 220.4 | 164.3 | 398.6 |
| NKp46.33 | IGHV3-23*03 | 64.6 | 73.8 | 0 | 1 | 0 | 1 | 0 | 6.4 | 214.3 | 147.7 | 344.6 |
| NKp46.34 | IGHV3-23*01 | 78.3 | 88.8 | 0 | 0 | 1 | 0 | 0 | 9.0 | 68.1 | 46.3 | 242.3 |
| NKp46.35 | IGHV3-66*01 | 63.4 | 70.0 | 0 | 0 | 3 | 1 | 0 | 7.1 | 129.7 | 67.2 | 267.2 |
| NKp46.36 | IGHV3-43*02 | 64.9 | 68.8 | 2 | 1 | 0 | 1 | 1 | 3.6 | 45.1 | 6.3 | 235.9 |
| NKp46.37 | IGHV3-9*02 | 64.3 | 70.0 | 1 | 1 | 1 | 0 | 0 | 4.2 | 124.5 | 101.4 | 431.2 |
| NKp46.38 | IGHV3-23*05 | 66.7 | 72.5 | 2 | 1 | 1 | 2 | 0 | 4.0 | 136.7 | 98.7 | 386.0 |
| NKp46.39 | IGHV3-23*02 | 64.0 | 71.3 | 0 | 1 | 2 | 1 | 0 | 9.2 | 115.0 | 61.2 | 580.5 |
| NKp46.40 | IGHV3-30*02 | 63.4 | 73.4 | 0 | 0 | 0 | 3 | 0 | 4.3 | 43.9 | 27.5 | 268.1 |

sdAbs were analyzed for their sequence identity compared to the most similar human germline (MOST SIMILAR GERMLINE) either based on the entire variable chain region (SEQ-ID) or the framework region only (SEQ-ID FW) as well as for their total number of specific chemical liabilities and PTMs, i.e., non-canonical cysteins (NON-CANONICAL CYS), methionine oxidations (MET OXIDATION), deamidations or isomerizations, and N-glycosylations, in structurally exposed CDR residues as derived from automatically generated homology models. As calculated physico-chemical developability descriptors (IN SILICO PHYSCHEM), structure-based pI values (pIFv 3D), the AggScores of the entire variable regions and the AggScores of CDR regions only (CDR AggScore), as well as the Positive Patch Energy of the CDRs (CDR Positive Patch Energy) are shown. The complementing color coding indicates scores within one standard deviation from a benchmark mean (dataset of 77 biotherapeutics approved for human application) as green, scores above one standard deviation as yellow and scores above two standard deviations as red. For the AggScores, this classification was slightly adjusted based on correlation analyses to internal experimental HIC data.


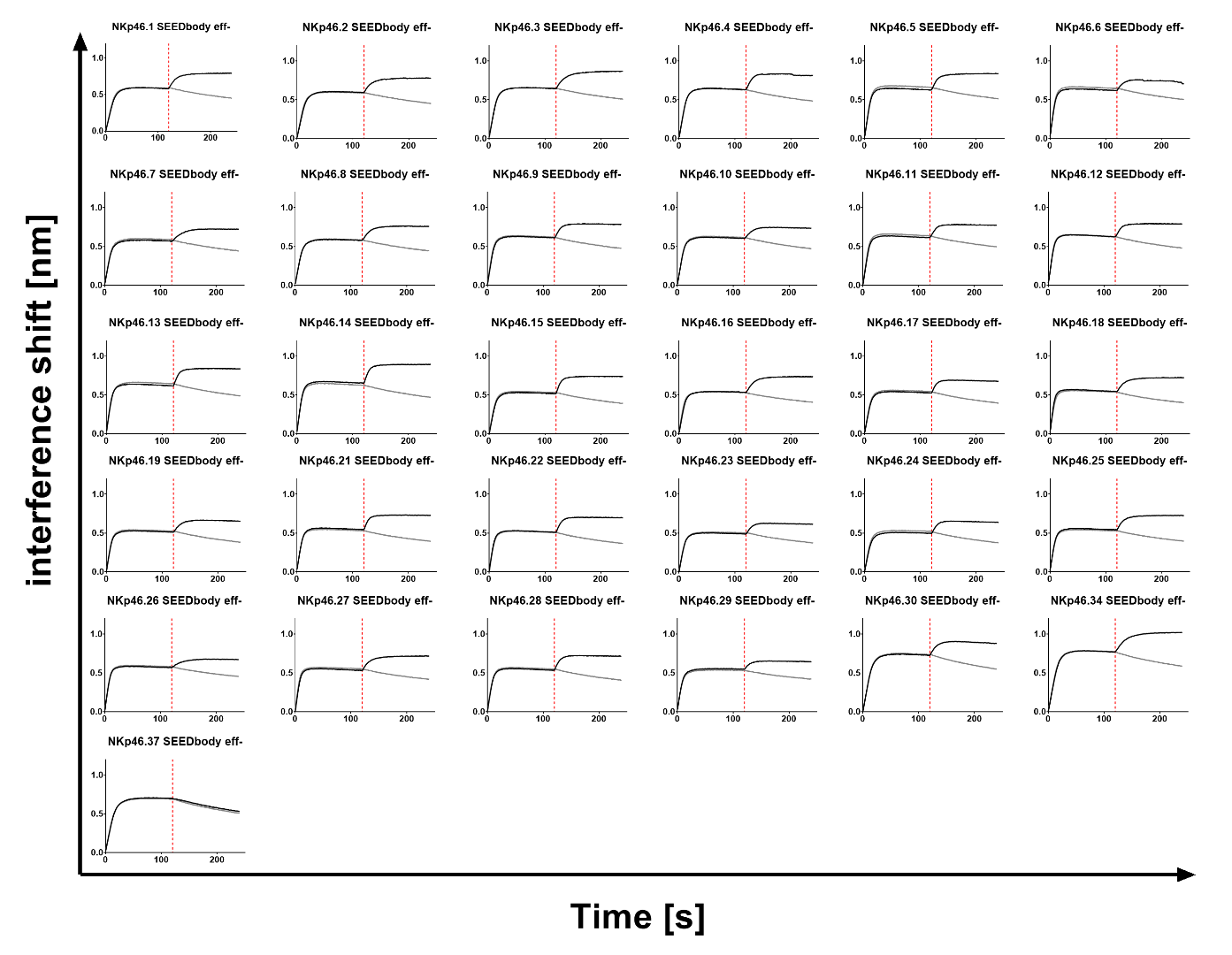


**Fig S1: NKp46 SEEDbodies demonstrate simultaneous antigen binding capacities in BLI assays.** Sensograms show simultaneous binding experiments to recombinant human NKp46 ECD as well as recombinant human EGFR ECD. EGFR was immobilized via its polyhistidine-tag to the sensor tip followed by a first association step using the respective SEEDbody at a concentration of 100 nM. Subsequently, a second association step was performed using rhNKp46 ECD at 100 nM. In each experiment KB buffer measurements were included as controls. Of note, NKp46.37 SEEDbody eff- is a NKp46 non-binding molecule and included as negative control for comparison.


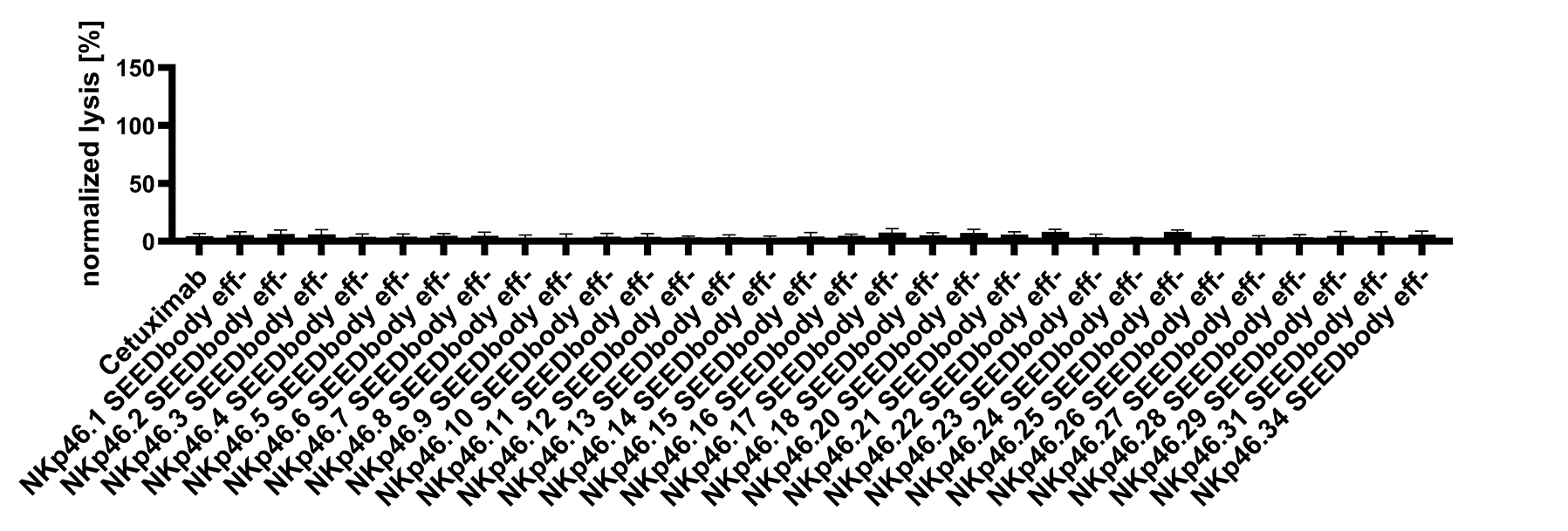


**Fig. S2: Generated bispecific NKp46 x EGFR SEEDbodies do not mediate significant lysis of EGFR-negative target cells.** Fluorescence-microscopy based killing assays with EGFR-negative ExpiCHO^TM^ target cells and freshly isolated PBMC-derived NK effector cells at an E:T ratio of 5:1. Analysis of target cell killing at 50 nM SEEDbody eff- concentration via NK cell-mediated ADCC. Reference molecule Cetuximab is included. Data was normalized to maximum tumor cell lysis mediated by 30 µM staurosporine to allow for comparison of independent experiments. Graphs show normalized means ± SEM of n = 4 different healthy donors.


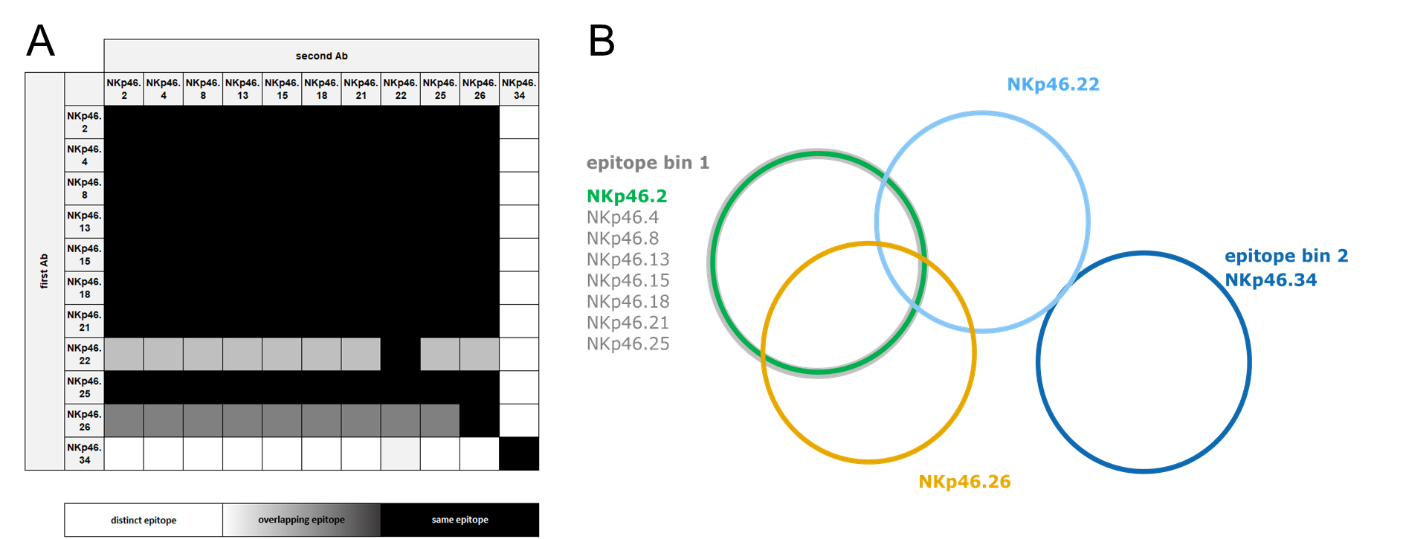


**Fig. S3: Pairwise competition assays via BLI revealed several different epitope bins for identified NKp46 specific sdAbs.** (A) Competition assays in every possible combination and orientation of the NKp46 VHH SEEDbodies enabled the identification of four epitope bins. Black boxes indicate competitive binding to NKp46, white boxes show non-competitive binding and grey boxes indicate partial competition, i.e., overlapping epitopes. (B) Epitope diagram deduced from the competition study. Epitopes are illustrated as circles. Distinct bins are indicated as bin 1 and bin 2. Overlapping circles represent (partially) competing antibodies.


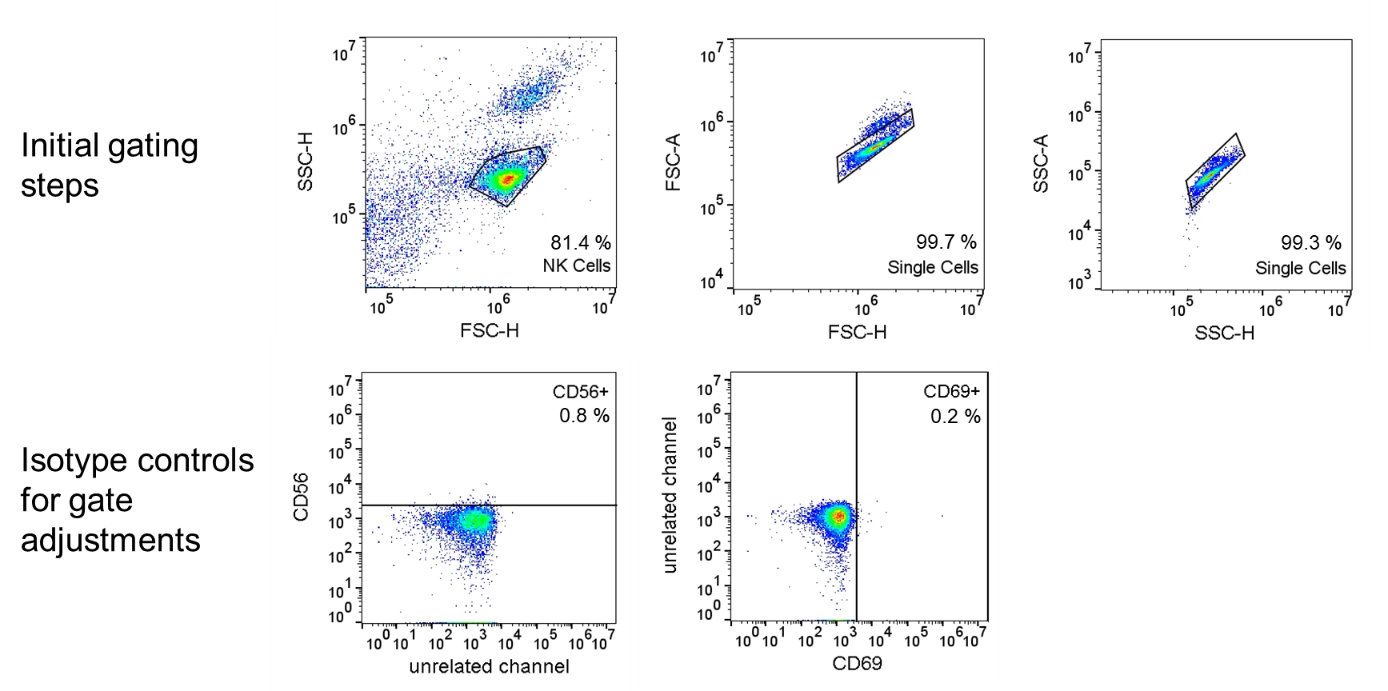


**Fig. S4: Sequential gating strategy enables CD69 detection on CD56-positive NK cells meditated by NKp46 x EGFR SEEDbodies.** Purified NK cells from PBMCs of healthy donors were incubated with EGFR-expressing A431 target cells (E:T = 5:1) at 37°C for 24 hours in the presence of 50 nM antibody prior staining with anti-CD56 and anti-CD69 antibodies and flow cytometric analysis. Plots in the upper row exemplarily demonstrate the gating strategy for single NK cell identification using forward and side scatter. Plots in the lower row show subsequent gating for CD56-positive cells (left) and CD69-positive cells (right) based on the depicted respective isotype controls. Each plot also includes respective percentage of gated cells.


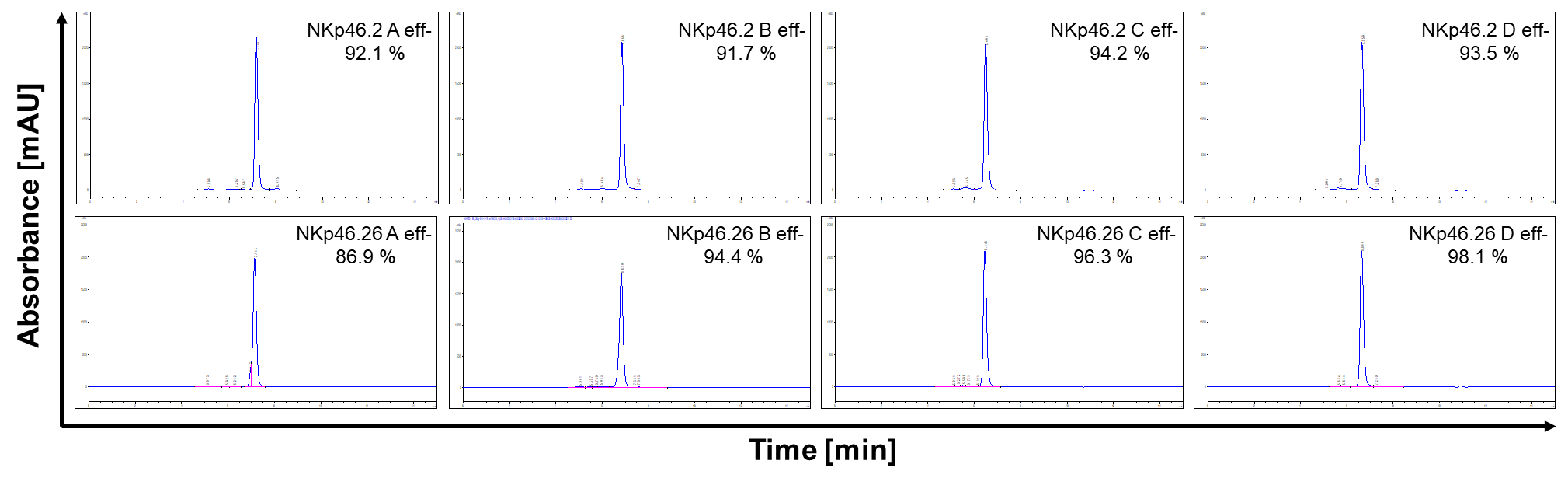


**Fig. S5: Size exclusion chromatography analysis unveils favorable purities post protein A purification of different VHH-based NKCE molecules.** SEC-HPLC profiles for different formats of NKp46.2 (upper row) and NKp46.26 (lower row)-based bispecific NKCEs as well as the percentage of the corresponding target monomer peaks are shown.


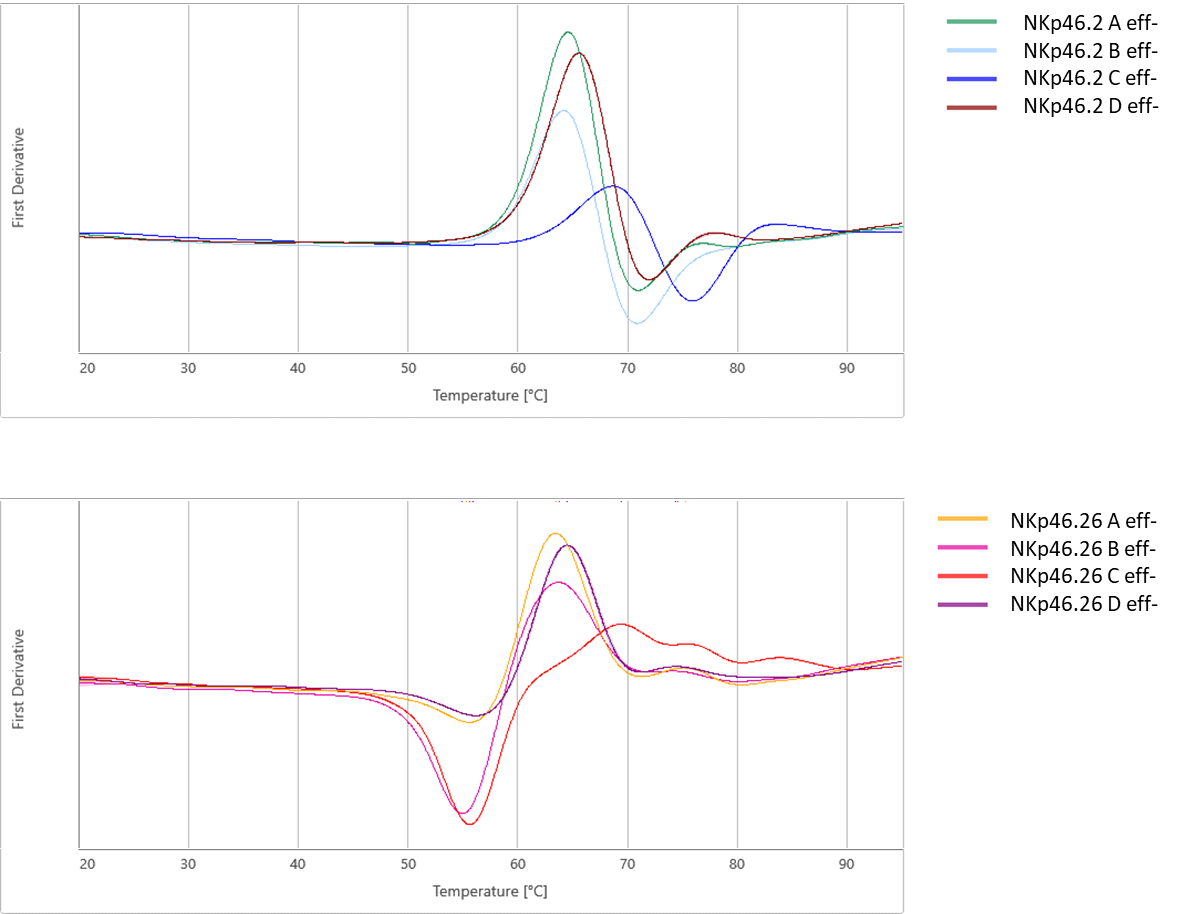


**Fig. S6: Thermal unfolding of NKp46 VHH-based SEEDbodies and IgG1 formats by differential scanning fluorimetry shows no negative influence of molecule architecture on thermal stabilities.** Overlays of the melting curves for different formats of NKp46.2 (upper panel) and NKp46.26 (lower panel)-based bispecific NKCEs were recorded utilizing a temperature gradient from 20°C to 95°C at a slope of 1°C/min. First derivatives of 350 nm/330 nm curves are shown.


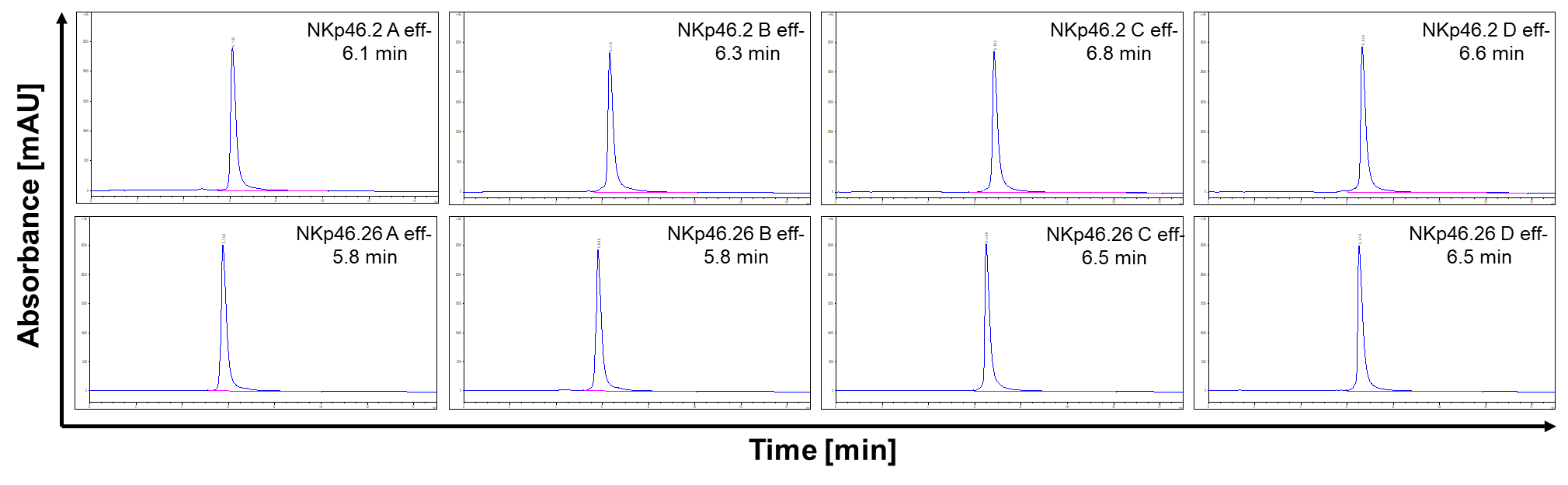


**Fig. S7: Hydrophobic interaction chromatography analysis shows format influences for NKp46 VHH-based NKCE molecules.** HIC profiles for different formats of NKp46.2 (upper panel) and NKp46.26 (lower panel)-based bispecific NKCEs as well as the corresponding retention times are shown.


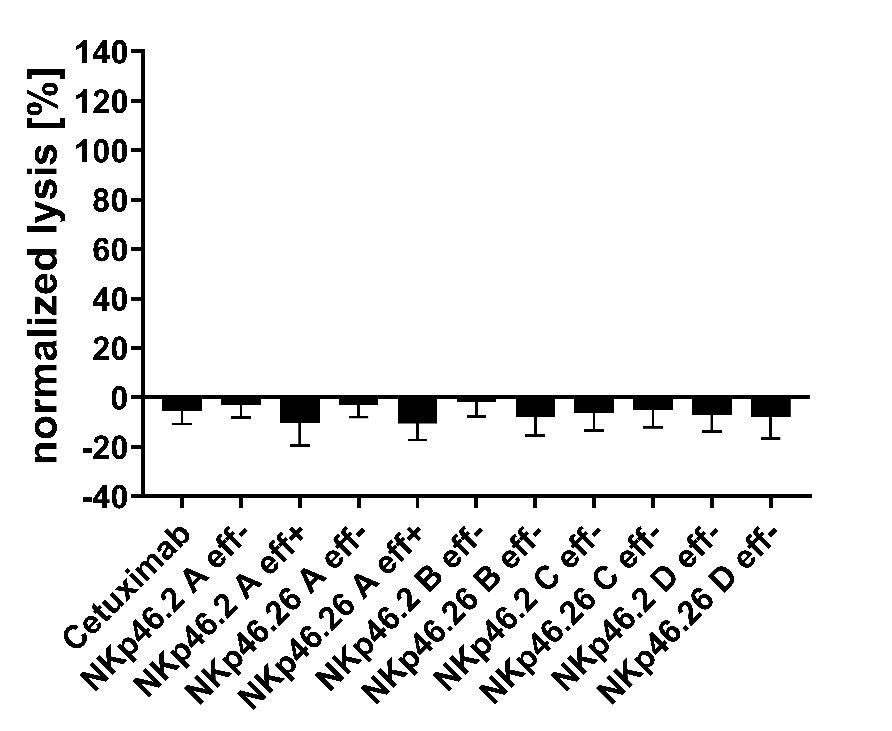


**Fig. S8: NKp46-targeting VHH-based NKCEs in different molecular architectures do not trigger significant cytotoxic activities of NK cells against EGFR-negative target cells.** Fluorescence-microscopy based killing assays with EGFR-negative ExpiCHO^TM^ target cells and freshly isolated PBMC-derived NK effector cells at an E:T ratio of 5:1. Analysis of maximum target cell killing at 50 nM antibody concentration for all scrutinized NKCE formats, based on NKp46.2 as well as NKp46.26 VHH. Reference molecule Cetuximab is included. Data was normalized to maximum tumor cell lysis mediated by 30 µM staurosporine to allow for comparison of independent experiments. Graphs show normalized means ± SEM of n = 8 different healthy donors.


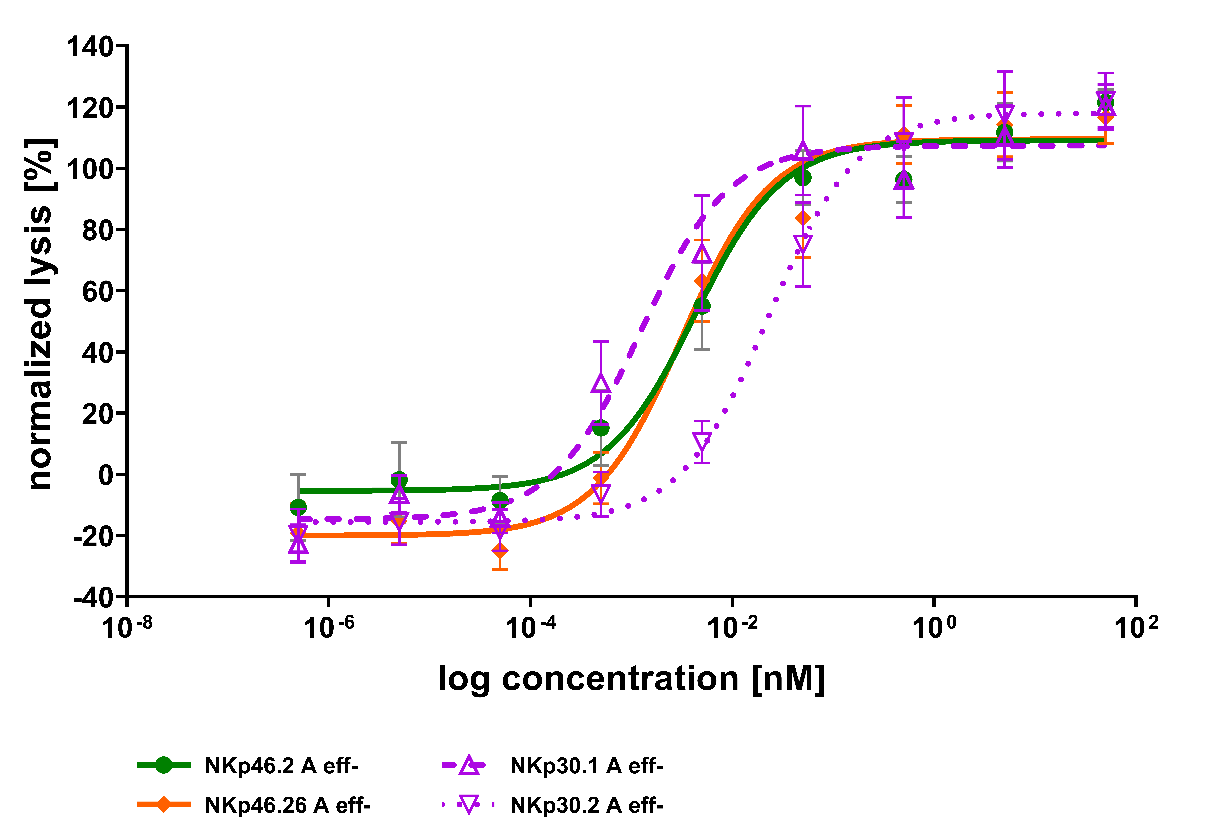


**Fig. S9: Effector silenced NKCEs based on either NKp46-specific or NKp30-targeting sdAbs elicit similar NK cell-mediated lysis of EGFR-overexpressing A431 cells.** Fluorescence based killing assays were conducted using A431 cells and freshly isolated PBMC-derived NK cells derived from healthy donors at an effector-to-target cell (E:T) ratio of 5:1. Strictly monovalent bispecific NKp46 or NKp30-specific VHH SEEDbodies harboring a humanized version of the Fab arm of Cetuximab as well as an effector-silenced Fc region were analyzed in a dose-dependent manner. Mean values ± SEM of seven independent experiments with biological duplicates are indicated. Data was normalized to the maximum concentration of Cetuximab to allow for comparison.
